# RS-CARES: Context-Aware Cross-Modal Alignment with Semantic Spatial Prior for Referring Remote Sensing Image Segmentation

**DOI:** 10.3390/jimaging12090453

**Published:** 2026-09-19

**Authors:** Hui Xiong, Wen Luo, Bing He

**Affiliations:** School of Computer Science, Chengdu University of Information Technology, Chengdu 610225, China

**Keywords:** referring image segmentation, vision–language model, cross-modal alignment, dynamic query, remote sensing interpretation

## Abstract

Remote sensing referring image segmentation faces critical challenges including arbitrary target rotation, drastic scale variation, cluttered complex backgrounds, and large visual-language semantic gaps. Existing mainstream segmentation models adopt fixed-receptive-field backbones, coarse unidirectional cross-modal interaction and static learnable object queries, which easily cause small-object missed detection, blurred boundary segmentation and fragmented predictions. This paper proposes a context-aware referring expression segmentation model for remote sensing to tackle the above limitations. We construct a dual-stream feature extraction backbone with InternImage and CLIP text encoder, and design a semantic prior localization map module to generate spatial heatmaps for spatial inductive bias and improve small-object localization recall. A cross-modal context aggregator performs multi-scale bidirectional visual-text alignment, while a dynamic query initialization strategy and a language-guided Transformer decoder progressively improve target localization and mask refinement. Experiments are conducted on two standard RRSIS benchmarks, RefSegRS and RRSIS-D. On RefSegRS, the proposed model achieves an mIoU of 70.43% and an oIoU of 77.34%, and obtains significant improvements on small vehicles, buildings and slender road markings. On the larger-scale RRSIS-D dataset, our model achieves an mIoU of 63.71% and an oIoU of 73.68%. The proposed model provides a solution for accurate multi-scale target segmentation guided by natural language descriptions in complex remote sensing scenes.

## 1. Introduction

With the rapid advancement of remote sensing satellites and aerial imaging technologies, the volume of acquired remote sensing imagery has increased exponentially. Efficiently and accurately extracting user-specified target information from massive remote sensing datasets has become a fundamental challenge in intelligent remote sensing image interpretation [1]. High-precision remote sensing image segmentation serves as a critical foundation for land surface analysis and scientific decision-making. However, conventional semantic segmentation methods based on predefined category sets can only recognize a limited number of fixed classes and lack the flexibility to locate and segment arbitrary targets described by users through natural language. In practical scenarios, users often need to retrieve and segment specific targets using natural language queries, motivating the emergence of the cutting-edge task of Referring Remote Sensing Image Segmentation (RRSIS) [2,3].

Compared with natural image segmentation tasks [4], remote sensing image segmentation presents more challenging scenarios. First, remote sensing images are typically acquired from a top-down perspective, where targets exhibit arbitrary orientations and substantial scale variations with differences in object size. Second, remote sensing scenes often contain complex backgrounds and densely distributed objects with similar visual appearances. Moreover, natural language queries frequently involve ambiguous references, complex spatial relationships, and multiple attribute constraints, further increasing the cross-modal semantic gap between visual and textual modalities. Therefore, effectively bridging the cross-modal semantic gap between complex linguistic descriptions and large-scale remote sensing scenes remains a critical scientific challenge for improving model performance.

The core challenge of RRSIS is to develop models that can simultaneously achieve accurate cross-modal semantic alignment and fine-grained object segmentation under increasingly complex remote sensing scenarios. Existing referring image segmentation methods can be broadly categorized into two groups: CNN-based fusion approaches and Transformer-based fusion approaches. Recent RRSIS methods, such as FIANet [5], have improved fine-grained image-text alignment. CNN-based methods [4,6] typically extract linguistic and visual representations separately using long short-term memory networks or convolutional neural networks, followed by simple feature concatenation or shallow attention-based interactions. Transformer-based methods [7,8] leverage self-attention mechanisms to enable deeper cross-modal feature interactions. However, these methods still exhibit substantial limitations when directly applied to remote sensing scenarios. First, at the visual feature extraction level, most existing methods adopt ResNet [9] or Swin Transformer [10] as backbone networks. However, their fixed receptive fields and axis-aligned local attention windows lack adaptability to geometric deformations and rotation variations, making them insufficient for handling extreme scale changes and arbitrary orientations of remote sensing objects. Second, at the cross-modal feature alignment stage, current approaches typically adopt coarse-grained and partially unidirectional interaction mechanisms that fail to deliver continuous linguistic guidance throughout the decoding process. Third, regarding target localization and query initialization, conventional Mask2Former [11] relies on purely learnable static embeddings as object queries. Such prior-free initialization lacks sufficient spatial guidance for small objects, potentially causing dispersed attention responses and missed targets. Fourth, in terms of boundary segmentation accuracy, remote sensing objects often exhibit complex and sharp geometric structures. Conventional segmentation heads suffer from feature degradation caused by interpolation-based upsampling, leading to blurred predictions around object boundaries. Fifth, from the perspective of training supervision, remote sensing objects exhibit characteristics including slender structures, large-scale variations, and densely distributed similar instances. Conventional supervision strategies that rely solely on query classification cross-entropy loss, BCE loss, and Dice loss [12] may result in fragmented predictions or boundary leakage, while lacking explicit constraints on local pixel-wise consistency.

In this context, we propose RS-CARES, a Context-Aware Referring Expression Segmentation framework for remote sensing. RS-CARES addresses the challenges of cross-modal alignment and small-object localization in complex scenes by combining a multi-scale visual backbone with frozen CLIP language representations. A Semantic Prior Localization Map (SPLM) provides expression-aware spatial priors for dynamic query initialization, while a Cross-modal Context Aggregator (CCA) enhances semantic interaction between language and multi-scale visual features. Based on these representations, a dynamic query module selects spatially diverse, expression-relevant candidates from high-resolution features. The resulting queries are further refined by a language-guided Transformer decoder for progressive target localization and mask prediction. These modules are integrated within a unified Mask2Former decoding framework, improving referring object segmentation in complex remote sensing scenes.

The main contributions of this paper are summarized as follows:

(1) We propose a CLIP-guided Semantic Prior Localization Map (SPLM) that constructs expression-aware spatial priors by combining frozen CLIP representations with multi-scale visual features. The resulting priors guide dynamic query selection and improve the localization of referring objects, particularly small targets.

(2) We design a Cross-modal Context Aggregator (CCA) that performs bidirectional vision–language interaction and cross-scale feature fusion, thereby enhancing semantic alignment and contextual modeling in complex remote sensing scenes.

(3) We develop a small-object-oriented dynamic query initialization strategy that combines global linguistic priors with high-resolution visual features. By incorporating expression-aware spatial priors and promoting spatial diversity, it generates more informative initial queries for referring object segmentation.

(4) We introduce a language-guided Transformer mask decoder that progressively integrates sentence-level and word-level linguistic information with visual features, improving target localization and mask prediction within a unified Mask2Former framework.

## 2. Related Works

### 2.1. Research on Remote Sensing Image Segmentation

Remote sensing image segmentation technology has gradually evolved from traditional handcrafted feature-based methods to deep learning approaches [13,14] and, more recently, vision–language models. Traditional methods, such as threshold-based segmentation and region growing, exhibit limited generalization capability and are difficult to adapt to complex remote sensing scenarios. With the development of deep learning, FCN [15] pioneered the end-to-end pixel-level segmentation paradigm. Subsequently, U-Net [16], DeepLab series [17], PSPNet [18], and SegNet [19] continuously improved the detail preservation and contextual modeling capabilities of remote sensing image segmentation through strategies including skip connections, atrous spatial pyramid pooling, multi-scale scene aggregation, and pooling index preservation, respectively.

Regarding backbone networks, ResNet has become a widely adopted feature extraction architecture due to its residual learning mechanism. However, the fixed convolutional structure is insufficient for adapting to the rotation variations and geometric deformations commonly existing in remote sensing targets. SENet [20], CBAM [21], and DANet [22] introduced attention mechanisms to enhance feature representation and long-range dependency modeling, but they still suffer from the limitation of fixed receptive fields. Swin Transformer [23] employs window-based self-attention to achieve hierarchical feature representation learning; however, its fixed window partition strategy cannot fully adapt to irregularly distributed geographic objects. InternImage [13], based on DCNv3 dynamic convolution, enables adaptive spatial sampling and effectively improves the modeling capability for complex geometric structures and rotated targets, achieving performance comparable to vision Transformers.

### 2.2. Research on Referring Remote Sensing Image Segmentation

Referring Image Segmentation (RIS) is an important multimodal vision task that aims to identify and segment target objects based on natural language descriptions. Hu et al. [4] first proposed this task and established a basic LSTM-CNN-based vision–language fusion framework. To improve the accuracy of cross-modal matching, early studies introduced a series of methods, including the Recurrent Refinement Network (RRN) [6], Cross-Modal Self-Attention (CMSA) [24], Language Structure Constraint Modeling (LSCM) [25], Cross-Modal Progressive Comprehension (CMPC) [26,27], and text grouping fusion strategies [28]. However, these CNN-based approaches generally suffer from limited receptive fields and insufficient cross-modal interaction capability.

The emergence of Transformer architectures has significantly advanced RIS by enabling more effective vision–language interaction. Methods such as VLT [29], LAVT [7], CRIS [30], CrossVLT [8], and ETRIS [31] have continuously improved RIS performance through multi-stage vision–language alignment, pretrained knowledge transfer, full-level bidirectional enhancement, and parameter-efficient fine-tuning strategies. Mask2Former [11] has become a widely adopted general segmentation framework and provides an effective query-based segmentation paradigm for RIS. Furthermore, Liu et al. [32] introduced the Generalized Referring Expression Segmentation (GRES) task and constructed the gRefCOCO dataset, further expanding the research boundary of referring segmentation from single-object segmentation to multi-object scenarios.

Referring Remote Sensing Image Segmentation (RRSIS) is an extension of RIS to remote sensing scenarios. Yuan et al. [3] first defined the RRSIS task and constructed the RefSegRS dataset. This study revealed the limitations of existing methods in segmenting small-scale and sparsely distributed remote sensing targets. Subsequently, Liu et al. [2] constructed the large-scale multi-resolution RRSIS-D dataset and proposed the RMSIN network, which utilizes scale interaction and adaptive rotation convolution to better characterize complex remote sensing objects. Although existing RRSIS studies have achieved considerable progress, several challenges remain unresolved. Specifically, current methods still suffer from insufficient adaptability of visual feature representations, incomplete bidirectional alignment between visual and language modalities, limited utilization of semantic priors, inaccurate boundary prediction for target objects, and the lack of local consistency constraints during training.

## 3. Methods and Materials

### 3.1. Methods

**Figure 1:**
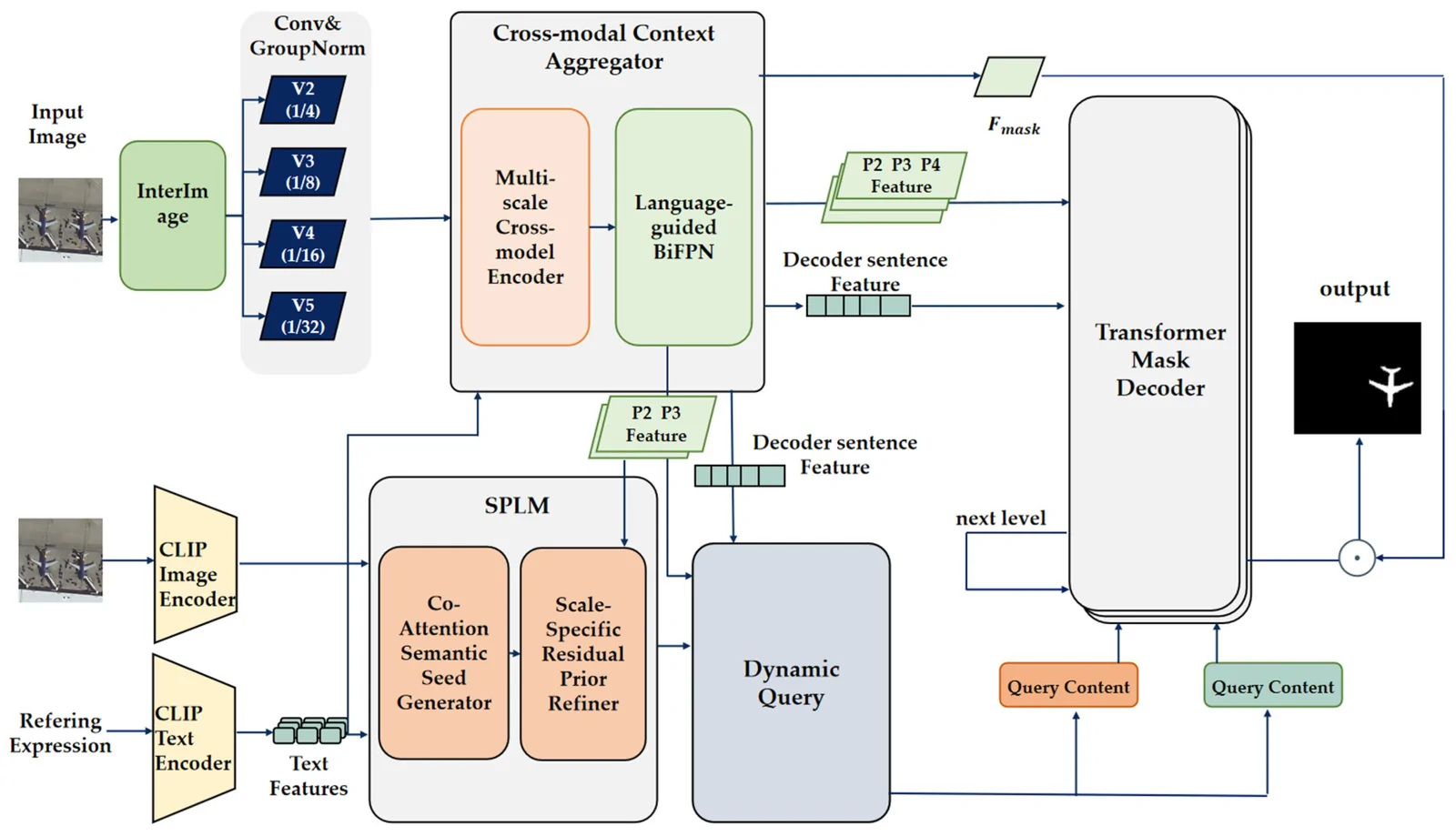
Architecture of the context-aware referring expression segmentation model for remote sensing.

The Context-Aware Referring Expression Segmentation model for remote sensing (RS-CARES) proposed in this paper consists of four core components: Basic Feature Extraction, Semantic Prior Localization and Dynamic Query Initialization, a Cross-modal Context Aggregator, and Language-guided Transformer Mask Decoding. The overall architecture of the model is illustrated in Figure 1.

As shown in Figure 1, the proposed network takes a remote sensing image and its corresponding referring expression as input. The visual branch utilizes InternImage to extract hierarchical visual features across four scales. Meanwhile, the text branch employs a frozen CLIP text encoder to obtain word-level and sentence-level linguistic representations. Simultaneously, the model leverages CLIP image and text representations to construct an expression-correlated Semantic Prior Localization Map (SPLM). This map provides the spatial conditions necessary for dynamic query initialization. Furthermore, the network extracts global visual context from the deepest visual features to supplement image-level scene information.

During the feature encoding stage, the three low-resolution visual scales first interact with word-level textual features through the Bidirectional Attention Transfer (BAT) module. Subsequently, they are fed into a Multi-Scale Deformable Attention (MSDeformAttn) encoder to aggregate visual contextual information across different spatial scales. The encoded deep visual features, along with the high-resolution shallow features retained from the backbone network, are then input into a language-guided Bidirectional Feature Pyramid Network (BiFPN). This pyramid fuses multi-scale visual information along both top-down and bottom-up pathways. At each fusion node, it continuously updates the linguistic representations, thereby enhancing cross-modal semantic consistency across features of various resolutions. Following the fusion process, the highest-resolution features are used to generate pixel-level mask representations, while the multi-scale outputs serve as the visual memory for the Transformer decoder.

During the decoding stage, the network integrates sentence-level semantic conditions with SPLM spatial priors to filter expression-relevant local candidate locations from the high-resolution P2 and P3 features. It then constructs dynamic object queries through cross-scale quota allocation and spatial deduplication. In each decoding layer, these initialized queries undergo language-guided context gating. They then sequentially perform cross-attention interactions with the multi-scale visual memory and word-level textual features, and are progressively updated via self-attention and feed-forward networks. Finally, the updated query representations are matched with the pixel-level mask features to directly generate the target segmentation masks, ultimately yielding the final referring expression segmentation results.

#### 3.1.1. Backbone Feature Extraction and Enhancement

During feature extraction, the network adopts a dual-stream architecture to process language and visual inputs separately. For the referring expression, a pretrained language model maps the text to a sequence of text embeddings $\left(T_{word}=[t_{1},t_{2},\ldots ,t_{L}]\in R^{L\times C}\right)$, where L denotes the sequence length and C denotes the projected feature dimension. Meanwhile, the code generates a padding mask from the valid tokens in the text sequence and uses text positional encodings to preserve the sequential positions of the tokens. The word-level features are then averaged along the sequence dimension to obtain the sentence-level text feature ${(t}_{\mathrm{sent}}=\frac{1}{L}\sum_{l=1}^{L}t_{l}\in R^{C})$, which represents the global semantics of the entire expression and is used for subsequent dynamic query initialization and language-guided feature enhancement in the decoder. To distinguish the initial encoding state from the subsequent decoder language conditions, the dynamic query initialization and language-guided feature enhancement use the decoder sentence-level features re-aggregated after updates by the CCA and BiFPN.

The proposed network adopts InternImage as the backbone feature extractor, whose hierarchical multi-scale representations exhibit strong robustness to geometric transformations, including rotation and deformation. To project multi-scale visual features into a unified semantic space, each feature map is independently processed using a 1 × 1 convolution followed by Group Normalization (GroupNorm) and projected into a 256-dimensional feature space, resulting in the feature set $V=\{V_{2},V_{3},V_{4},V_{5}\}$.

Given that remote sensing images typically feature expansive fields of view and complex backgrounds, target objects often occupy only a fraction of the pixels. The zero-initialization or static learnable object queries used in traditional Transformer decoders tend to cause attention diffusion. Consequently, the model becomes difficult to train and highly susceptible to interference from complex backgrounds. To provide expression-relevant spatial inductive biases, we introduce a Semantic Prior Localization Map (SPLM) based on frozen CLIP image and text features. Instead of directly outputting it as the final segmentation mask, we utilize this map as auxiliary information to score dynamic query candidates.

First, the SPLM constructs frozen CLIP semantic seeds. After applying CLIP-consistent preprocessing to the original image and resizing it to 224 × 224, the frozen CLIP image encoder generates patch tokens, while the frozen CLIP text encoder generates word-level tokens. Both types of tokens reside within the CLIP projection space, and the text padding positions are explicitly masked in subsequent attention operations.

To adapt to the referring segmentation task while preserving the pre-trained alignment geometry, the SPLM introduces a residual bidirectional cross-attention mechanism between the two types of tokens. Specifically, the text tokens first query the patch tokens, and then the updated text tokens guide the update of the patch tokens. The two residual scaling coefficients are initialized to 0.1. Consequently, the initial behavior of the module closely approximates the original CLIP vision–language alignment, and only the attention adapters and their scaling coefficients are updated during training.

Specifically, the process takes the frozen CLIP image patch features $Z_{v}$ and the word-level textual features of the entire expression $Z_{t}$ as inputs. It then updates both types of tokens via residual bidirectional cross-attention:

(1) $$\begin{array}{c} {\tilde{Z}}_{t}=Z_{t}+\alpha_{t}MHA\left(Z_{t},z_{v},z_{v}\right),\;\\ {\tilde{Z}}_{v}=z_{v}+\alpha_{v}MHA(z_{v},{\tilde{Z}}_{t},{\tilde{Z}}_{t}) \end{array}$$

Here, $\alpha_{t}$ and $\alpha_{v}$ are learnable residual coefficients.

The module extracts the last valid text token to serve as the global semantic vector. It then performs normalized cosine matching between this vector and the updated patch features to generate the CLIP semantic seed map:

(2) $$L_{\mathrm{seed}}(p)=\frac{{\bar{z_{v}}(p)}^{T}\bar{z_{eot}}}{\tau },M_{seed}=\sigma (L_{seed}).$$

Here, $p\in \Omega_{c}$ denotes the spatial position within the coarse-grained patch grid output by the CLIP image encoder. $z_{v}(p)\in R^{D}$ represents the visual patch feature at position p after being updated by the residual bidirectional cross-attention. $z_{eot}\in R^{D}$ represents the feature of the valid EOT token at the end of the updated text sequence, which is used to encode the global semantics of the entire referring expression. The overline indicates $L_{2}$ normalization; thus, the numerator represents the cosine similarity between the visual patch and the expression semantics. D is the CLIP embedding dimension, τ>0 is the temperature coefficient, and σ(⋅) is the Sigmoid function. $L_{seed}(p)$ refers to the matching logits at position p, and $M_{seed}$ is the normalized semantic seed map composed of responses from all positions.

Because the CLIP patch grid is relatively coarse, the seed map can only provide a rough target region. This makes it difficult to directly satisfy the localization requirements for small object queries. Therefore, the network first bilinearly aligns the seed map to the spatial dimensions of $P_{2}$ and $P_{3}$ and then utilizes two parameter-independent residual localizers to perform scale-specific refinement. For any given scale i∈{2,3}, the localizer first projects the current-scale visual features into a hidden space using a 1×1 convolution. Simultaneously, it leverages the decoder sentence-level textual features—which are shared with the dynamic queries and LGC—and transforms them into channel gating weights via a linear mapping and a Sigmoid function to perform channel-wise modulation on the visual features.

Subsequently, the localizer concatenates the language-modulated visual features with the coarse prior logits (obtained by transforming the aligned seed map) along the channel dimension. It then predicts the prior residual through a sequence of operations including a 3×3 convolution, GroupNorm, GELU activation, and a 1×1 convolution:

(3) $$\begin{array}{c} \Delta L_{i}=R_{i}({Conv}_{1\times 1}(V_{i})⊙\sigma (W_{i}s_{dec})∥logit({\tilde{M}}_{i}))\\ L_{i}=logit\left({\tilde{M}}_{i}\right)+\Delta L_{i},\;\;M_{i}=\sigma (L_{i}) \end{array}$$

Here, $V_{i}$ denotes the visual features at the current scale. The term $s_{dec}$ represents the decoder sentence-level textual features. These features are aggregated after being updated by the CCA and the language-guided BiFPN. ${\tilde{M}}_{i}$ is the CLIP seed map aligned to the current scale. The symbol ∥ indicates concatenation along the channel dimension. $R_{i}$ represents the residual prediction branch.

The weights and biases of the 1×1 convolution at the end of the localizer are both initialized to zero. Therefore, the residual $\Delta L_{i}$ is approximately zero during the early stages of training. This allows the refined results to initially maintain the responses of the original CLIP seeds.

During the training phase, a localization loss supervises the refined priors that actually participate in sorting the $P_{2}$ and $P_{3}$ candidates. Specifically, the ground truth mask is first resized to the corresponding grid and converted into a Gaussian target. Subsequently, a soft Dice loss is calculated.

The refined $P_{2}$ and $P_{3}$ spatial prior maps do not directly replace the visual features. Instead, they work jointly with the vision–language similarities at their corresponding scales to rank the dynamic query candidates. Furthermore, they are combined with spatial deduplication to retain dispersed yet relevant positions. The localization constraints and list-wise ranking constraints applied during the training phase further guide the high-response candidates to fall within the referring target regions.

In addition, the network extracts a global visual context vector $v_{g}$ from the deep visual features. This vector supplements the image-level scene information that dynamic local queries struggle to cover. Specifically, the network takes the deepest features $V_{5}$ output by InternImage. It first projects them to the query feature dimension using a learnable 1×1 convolution. Then, it applies global average pooling across the spatial dimensions:

(4) $${F_{g}}^{\left(b\right)}={Conv}_{1\times 1}\left({V_{5}}^{\left(b\right)}\right),\;\;{v_{g}}^{\left(b\right)}=GAP({F_{g}}^{\left(b\right)})$$

Here, $V_{5}^{\left(b\right)}$ and $F_{g}^{\left(b\right)}$ denote the deepest visual features and their projection results for the b-th sample, respectively. The acronym GAP represents spatial global average pooling. Finally, $v_{g}$ is expanded from a shape of [B,C] to [1,B,C]. It is then added to the content initialization of each query.

$v_{g}$ is derived from the global aggregation of the deepest visual features. It is not involved in textual condition modulation. Furthermore, it does not belong to the SPLM spatial prior map. Its sole responsibility is to supplement the image-level scene information that is difficult for dynamic local queries to capture.

#### 3.1.2. Cross-Modal Context Aggregator

**Figure 2:**
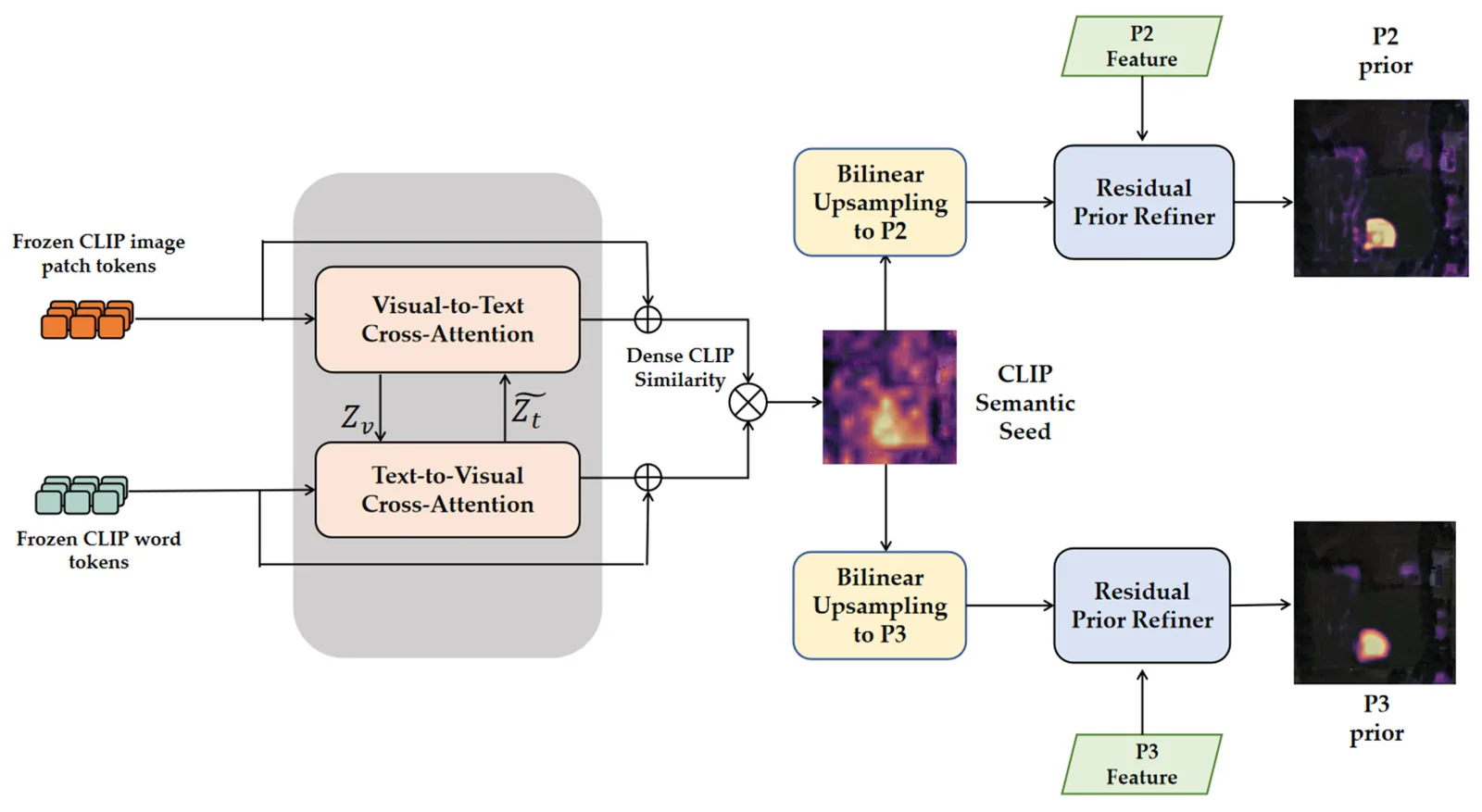
Overall architecture of the SPLM.

The primary objective of the Cross-Modal Context Aggregator (CCA) module (as shown in Figure 2) is to establish deep bidirectional interactions between visual and linguistic representations across multiple feature scales. Given the four-scale visual feature maps $\left\{V_{2},V_{3},V_{4},V_{5}\right\}$ extracted by the backbone network, with spatial strides of 4, 8, 16, and 32 pixels, respectively, together with the word-level textual representations $T_{word}\in R^{L\times B\times C}$ generated by the CLIP text encoder, the CCA module adopts a progressive multi-stage cross-modal fusion architecture to achieve semantic alignment while capturing rich multi-scale contextual information.

During the initial feature interaction stage, an early fusion strategy combining Bidirectional Attention Transfer (BAT) and Multi-Scale Deformable Self-Attention (MSDeformAttn) is applied to the three lower-resolution feature maps $V_{3}$, $V_{4}$ and $V_{5}$.

First, the visual feature map $V_{i}$ at each scale is independently fed into the BAT module for cross-modal interaction. The BAT module consists of two consecutive multi-head cross-attention stages. In the first stage, the textual representations serve as the queries (Q), whereas the visual features act as the keys (K) and values (V).

(5) $$T_{lang}=MHA(T_{word},V_{i},V_{i})⊙T_{word}$$

This operation generates a visually enhanced textual representation $T_{lang}$, enabling the language features to attend to semantically relevant regions within the current visual receptive field. In the second stage, $T_{lang}$ is employed as both the keys and values, while the original visual features serve as the queries.

(6) $$V_{i}^{\prime }=MHA(V_{i},T_{lang},T_{lang})⊙V_{i}$$

This operation performs language-guided visual feature refinement by suppressing background responses unrelated to the textual description, producing the enhanced visual representation $V_{i}^{\prime }$.

Subsequently, the BAT-enhanced features from the three scales are flattened, concatenated, and fed into a MSDeformAttn encoder. Compared with conventional Transformer encoders, MSDeformAttn computes attention only over a small set of sparse sampling locations around each reference point, substantially reducing the computational cost of multi-scale feature processing while producing the encoded feature set $\left\{V_{3}^{\prime \prime },V_{4}^{\prime \prime },V_{5}^{\prime \prime }\right\}$.

**Figure 3:**
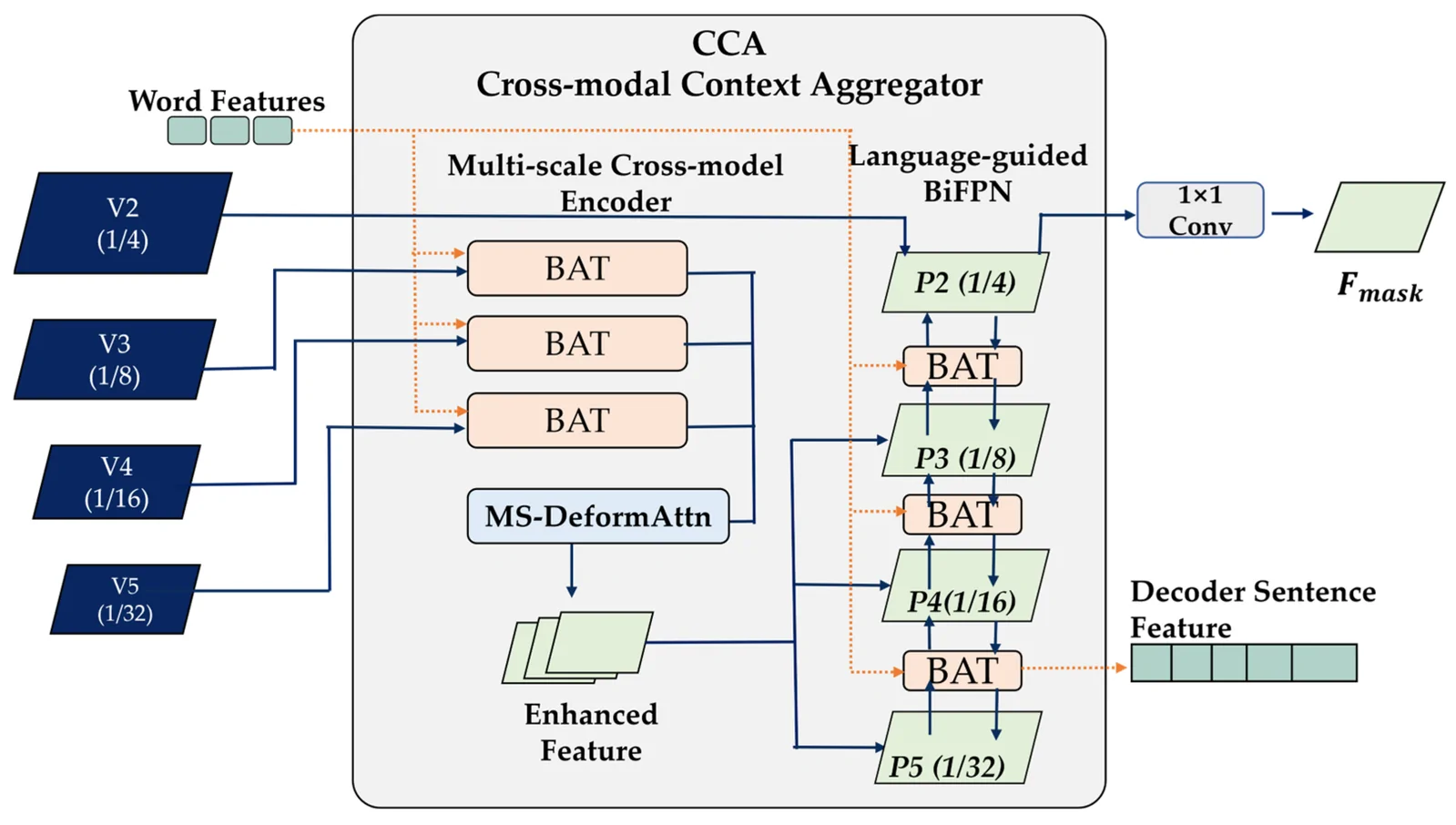
Architecture of the cross-modal context aggregator.

Following multi-scale feature encoding, a language-guided bidirectional feature pyramid network (BiFPN) is introduced to recover high-resolution spatial details and construct feature representations suitable for dense pixel-level prediction. The BiFPN takes the three encoded feature maps together with the stride-4 shallow feature retained from the backbone through lateral projection, thereby forming a four-level feature pyramid. Each input branch is first aligned to the target resolution, followed by weighted feature fusion using non-negative normalized learnable weights. The fused features are subsequently refined using a 3 × 3 convolution, normalization, and a ReLU activation function.The overall architecture of the cross-modal context aggregator, including the language-guided BiFPN, is illustrated in Figure 3.

Specifically, the network first sequentially generates $P_{4}^{td},\;\;P_{3}^{td}$, and $P_{2}^{out}$ along the top-down pathway. Then, it generates $P_{3}^{out},{\;P}_{4}^{out}$, and $P_{5}^{out}$ along the bottom-up pathway. All six scale fusion nodes incorporate the BAT module. The word-level textual features output by each node are passed to the next node. This allows the linguistic prompts to continuously participate in the refinement of visual features at different resolutions.

After the fusion is complete, the highest-resolution feature $P_{2}^{out}$ is projected via a 1 ×1 convolution to generate high-resolution pixel-level mask representations. These representations are used for the subsequent dot product between the query mask embeddings and the pixel features. Simultaneously, $\left\{P_{2}^{out},P_{3}^{out},P_{4}^{out}\right\}$ are fed into the Transformer decoder to serve as three-scale visual memory. In contrast, $P_{5}^{out}$ only participates in the cross-scale fusion within the pyramid. It is not directly used as an input to the current decoder.

Furthermore, the final word-level textual features output by the BiFPN, along with their padding masks and positional encodings, continue to be passed to the subsequent layers of the pixel decoder. The network then aggregates these final word-level features to form the decoder sentence-level textual features $s_{dec}$. These sentence-level features act as global linguistic conditions. They are utilized for dynamic query initialization and the language gating of the LGC Gate in each decoding layer. The corresponding word-level textual features serve as the keys and values for the textual cross-attention.

#### 3.1.3. Dynamic Query Initialization for Small Object Segmentation

**Figure 4:**
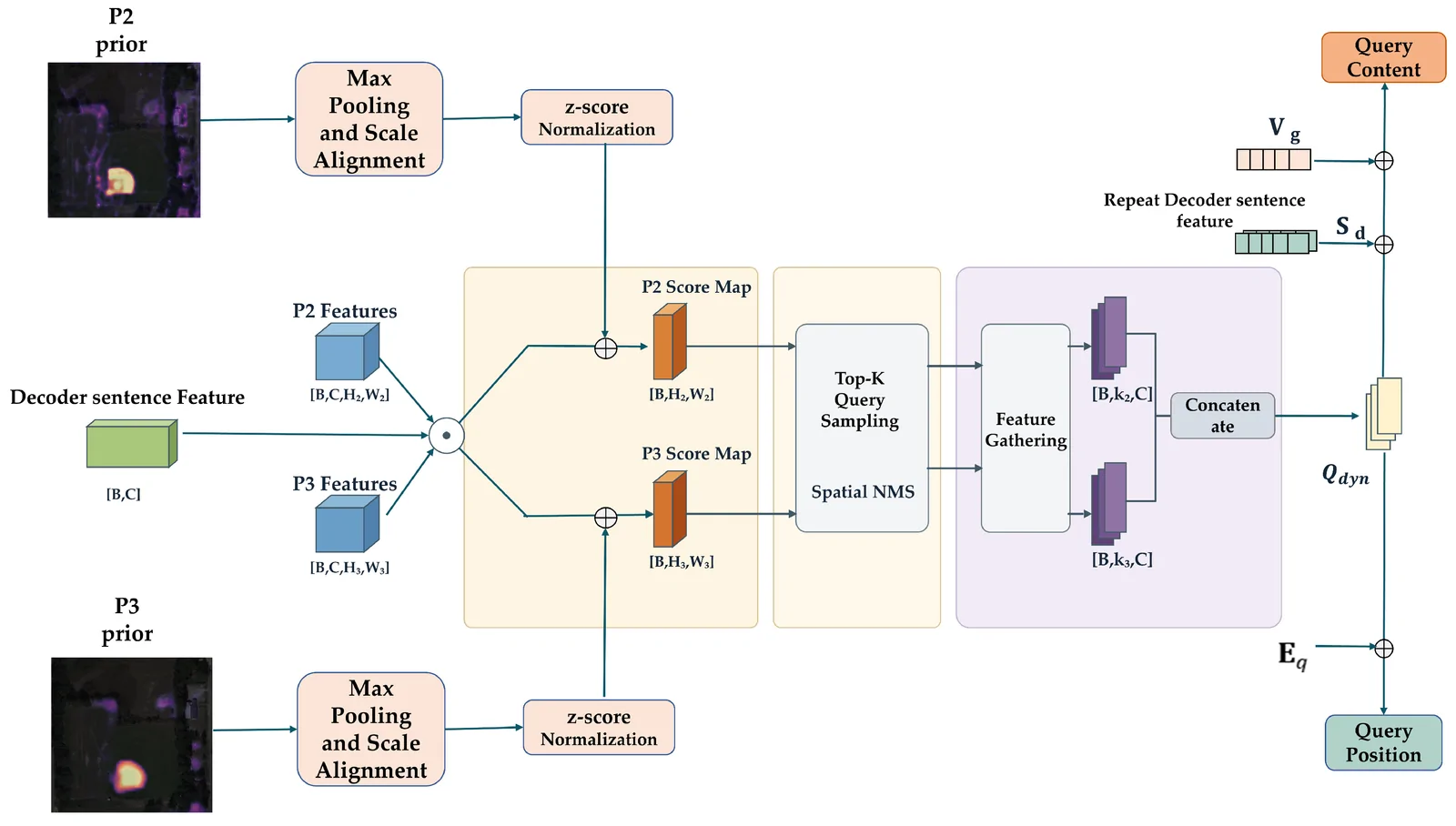
Architecture of dynamic query initialization.

During the mask prediction stage, we construct the decoding framework based on Mask2Former. In remote sensing images, referring targets are often surrounded by expansive backgrounds. Furthermore, small objects are easily weakened within high-level semantic features. If the Mask2Former decoder is directly initialized with fixed learnable vectors, the queries lack clear search regions during the initial stage. Consequently, they must rely on subsequent attention mechanisms to gradually filter out irrelevant backgrounds. To address this, we retain the standard iterative method of the mask decoder. Instead of changing it, we generate object queries from two high-resolution visual memories based on the current expression before they enter the decoder. This ensures that the queries already carry local visual evidence and expression-relevant spatial guidance prior to the first cross-attention operation.The architecture of the dynamic query initialization module is illustrated in Figure 4.

After being updated by the CCA and the language-guided BiFPN, we denote the visual memories with strides of 4 and 8 used for dynamic queries as $P_{2}$ and $P_{3}$. The final word-level textual features are aggregated along the sequence dimension to obtain the shared decoder sentence-level textual features $s_{dec}$. $s_{dec}$ is simultaneously used for $P_{2}$ and $P_{3}$ candidate ranking, SPLM refinement, and query content initialization. We no longer introduce mutually independent ranking sentence vectors, query sentence vectors, or ranking textual features. The SPLM outputs the refined $P_{2}$ and $P_{3}$ spatial prior maps $M_{i}$. The visual memory $P_{i}\in R^{B\times C\times H_{i}\times W_{i}}$ has a strict one-to-one correspondence with $M_{i}$ in terms of spatial dimensions. Here, i∈{2,3}.

Let ${\bar{P}}_{i}$ denote the candidate feature map at the i-th scale, where i∈{2,3}. Let $s_{dec}$ represent the sentence-level semantic embedding, and let $M_{i}$ denote the spatial semantic prior aligned to the corresponding feature scale. For each spatial location p, a candidate confidence score is computed as follows:

(7) $$\begin{array}{c} S_{i}(p)=\frac{{\bar{P}}_{i}(p)}{\|{\bar{P}}_{i}(p){\|}_{2}}\cdot \frac{s_{dec}}{\|s_{dec}{\|}_{2}}+\lambda_{s}\sigma (g)\;clip\left(\frac{M_{i}(p)-\mu_{i}}{\sigma_{i}+\varepsilon },-3,3\right),\\ i\in \{2,3\} \end{array}$$

Here, “⋅” denotes the dot product between the normalized candidate feature vector and the normalized sentence-level semantic embedding. Here, $\mu_{i}$ and $\sigma_{i}$ denote the spatial mean and standard deviation of the spatial prior map $M_{i}$ at the i-th candidate scale for the current sample, respectively. The term g is a learnable gating parameter. $\lambda_{s}$ represents the prior weight, which is set to 1.0 in the experiments. To eliminate differences in the amplitude and dispersion of prior responses across different samples and scales, the model first performs z-score standardization on $M_{i}$ along the spatial dimensions:

(8) $$Z_{i}(p)=\frac{M_{i}(p)-\mu_{i}}{\sigma_{i}+\varepsilon }$$

Here, ε is a tiny constant introduced to prevent numerical instability when the standard deviation is too small. Subsequently, the model clips the standardized results to the interval [−3,3], denoted as $\mathrm{clip}(Z_{i}(p),-3,3)$. This operation suppresses the excessive influence of a few extreme responses on the candidate ranking. Finally, the prior term modulated by σ(g) is added to the vision–language similarity score with a weight of $\lambda_{s}\sigma (g)$. This processing is solely used to calibrate the relative contribution of the prior map during candidate scoring. It is not directly used as the final segmentation mask.

This score primarily relies on the cosine similarity between the normalized visual features and textual features. It is then supplemented by the spatial prior that has undergone z-score standardization, clipping, and gating modulation. Consequently, this prevents uncalibrated dot products and overly strong spatial priors from interfering with the candidate ranking.

Subsequently, the model selects $k_{2}=round(0.6Q)$ and $k_{3}=Q-k_{2}$ candidate locations from the score maps of the two scales, respectively. Here, Q is set to 5. To reduce repetitive sampling in local regions, the candidate selection employs spatial deduplication based on normalized coordinate distances. This process is known as Spatial Non-Maximum Suppression (Spatial NMS). The normalized Euclidean distance between the selected coordinates in P2 must be no less than 0.04. Furthermore, the distance between the P3 candidates and the selected P2 coordinates must also meet this threshold. This strategy reduces the risk of multiple queries concentrating on the same salient location. Based on the selected coordinates, the model collects local visual vectors from the corresponding aggregated feature maps. It then concatenates them to form the dynamic query $Q_{dyn}$. Let $s_{dec}$ denote the decoder sentence-level semantic features. Let $v_{g}$ denote the global visual context vector obtained from deep visual features through projection and global average pooling. Let $E_{q}$ represent the learnable query positional embeddings. The query content and query positions are constructed as follows:

(9) $$Q_{content}={Repeat}_{Q}(s_{dec})+Q_{dyn}+v_{g},\;Q_{pos}=E_{q}+0.5\;Q_{dyn}$$

During the training process, the refined P2 and P3 prior maps are optimized through localization constraints and list-wise ranking constraints. This enables them to indicate the target regions more accurately. The specific loss definitions are detailed in Section 3.1.5.

It must be emphasized that the SPLM spatial prior maps only participate in the scoring and sampling of the P2 and P3 candidate locations. The global visual context vector $v_{g}$ is derived from the global aggregation of deep visual features. It is used to supplement the image-level scene information for the dynamic local queries. Therefore, the spatial prior maps and the global visual context vector serve different functions during query initialization. The former provides the basis for ranking local candidates. The latter supplements the image-level scene information. The dynamic bias in the query positional encodings comes from the local visual queries collected after the prior-assisted filtering. Consequently, the initialized object queries simultaneously possess expression-relevant semantic anchors, local spatial guidance oriented toward small objects, and global visual context. This provides a more reliable initial state for the cross-modal attention interactions in the subsequent Transformer decoder.

#### 3.1.4. Language-Guided Transformer Mask Decoding

**Figure 5:**
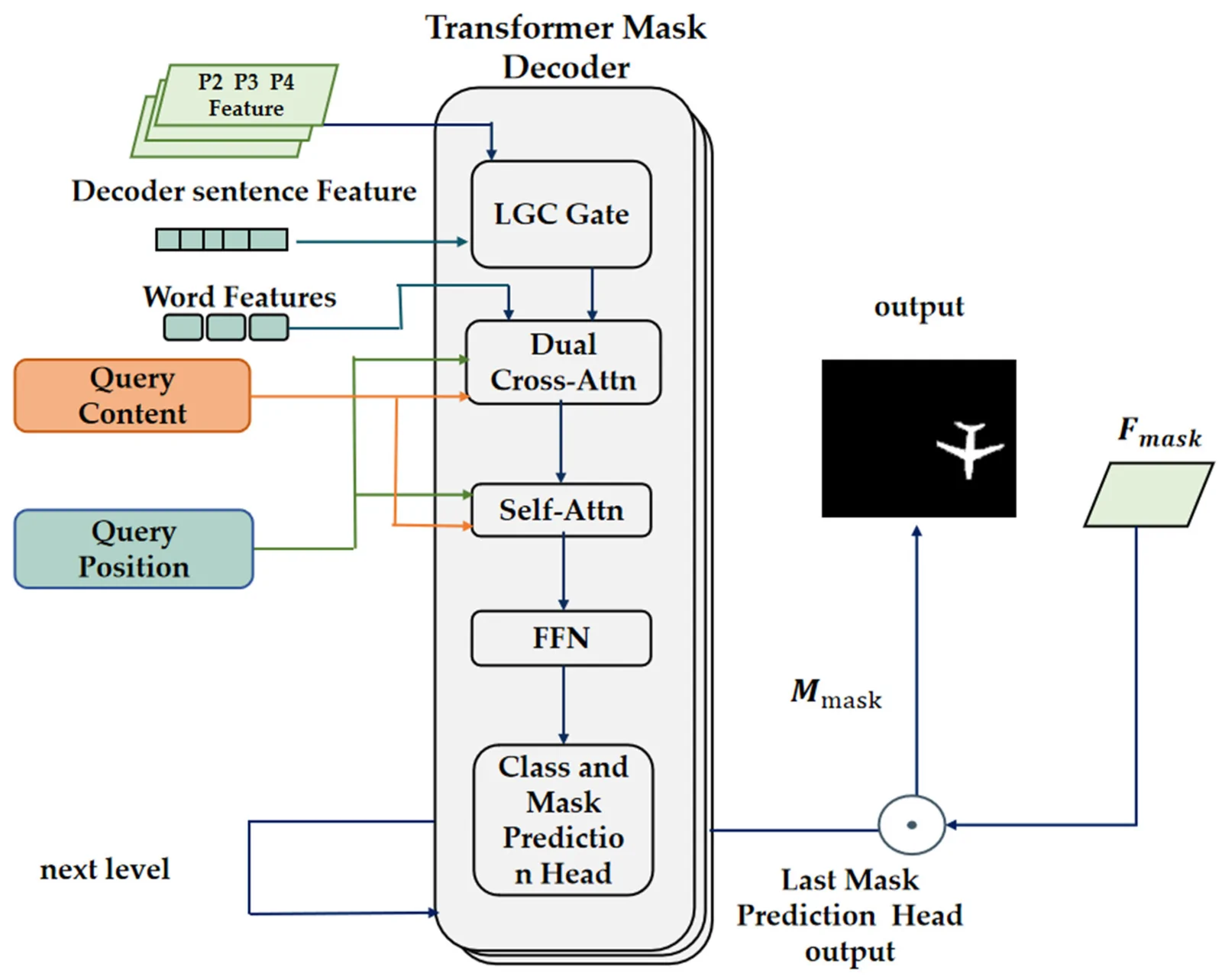
Framework of the language-guided Transformer mask decoding mechanism.

To progressively focus on target regions that align with the referring expression within complex remote sensing backgrounds, we construct a language-guided Transformer mask decoding module (as shown in Figure 5). This module takes the dynamically initialized queries as its starting point. It cyclically aggregates regional evidence from the multi-level visual memory across different scales. Simultaneously, it introduces fine-grained linguistic constraints from the word-level textual memory. Furthermore, sentence-level semantics modulate the current visual features prior to the visual cross-attention operation.

The module performs a layer-wise iteration consisting of language-guided visual enhancement, dual-path cross-attention, query self-attention, and feed-forward updates. Through this iterative process, the query representations progressively achieve cross-modal alignment and object relationship modeling. Ultimately, they generate the final segmentation masks corresponding to the referring expressions.

**Figure 6:**
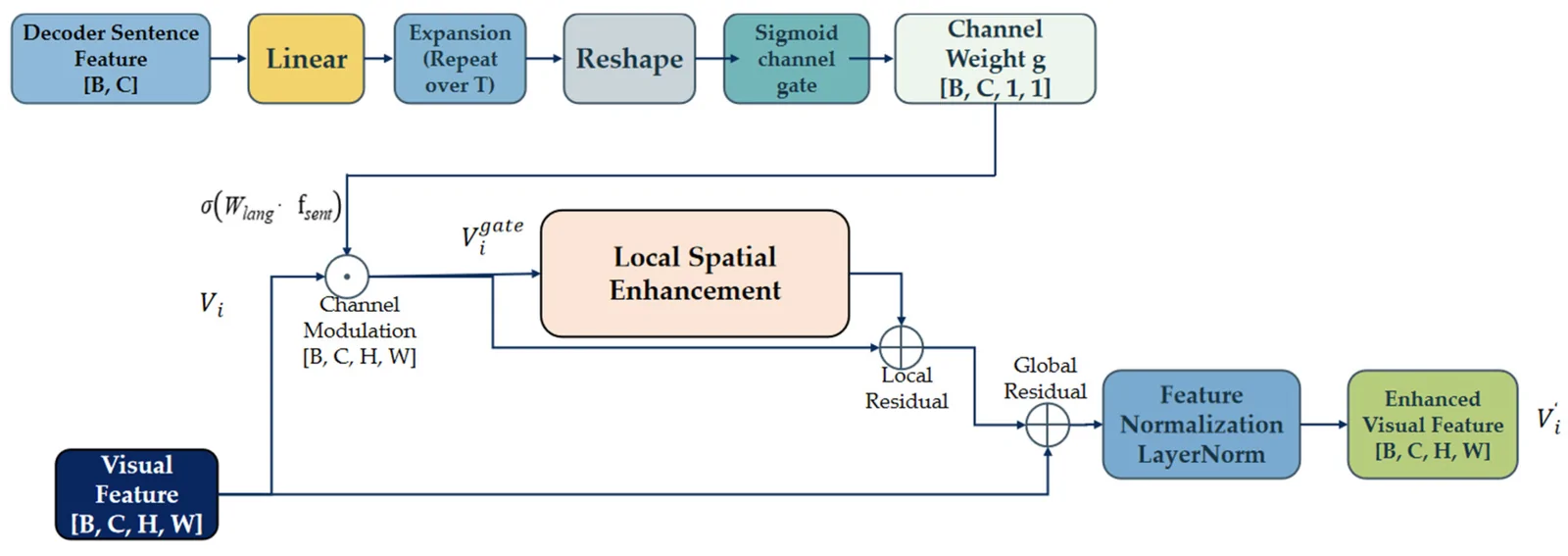
Architecture of the language-guided global context gating module.

After obtaining the dynamic queries and constructing the initial query representations, a Language-Guided Global Context Gating (LGC Gate) module is introduced before the visual cross-attention operation in each Transformer decoder layer, as illustrated in Figure 5. This module is designed to suppress interference from complex remote sensing backgrounds during cross-modal matching.The detailed architecture of the LGC Gate is shown in Figure 6.

Given the visual feature map $V_{i}$ at the current scale, the module generates channel-wise modulation weights conditioned on the sentence-level semantic representation $f_{sent}$.

(10) $$V_{i}^{gate}=V_{i}⊙\sigma \left(W_{lang}\cdot f_{sent}\right)$$

Building upon the channel-wise modulation, the module further employs a 3 × 3 depthwise convolution to capture local spatial contexts within each channel. A residual connection is introduced to preserve the original visual information, producing the enhanced visual representation:

(11) $$V_{i}^{\prime }={LN}_{c}\left(V_{i}+V_{i}^{gate}+{DWConv}_{3\times 3}(V_{i}^{gate})\right)$$

Here, ${\mathrm{LN}}_{C}$ denotes layer normalization performed along the channel dimension at each spatial position. The weights and biases of the depth-wise convolution are both initialized to zero. This ensures that the local spatial enhancement branch does not introduce additional random local perturbations during the early stages of training. Consequently, this helps maintain the stability of the decoding process.

Subsequently, the object queries perform cross-attention interactions simultaneously with the language-guided visual features and the word-level textual features. Here, Q represents the current query content. $Q_{\mathrm{pos}}$ denotes the query positional encodings. $T_{\mathrm{word}}$ represents the word-level textual feature sequence updated by the text encoder and the front-end vision–language interaction module. $E_{i}$ and $E_{T}$ represent the visual and textual positional encodings, respectively. $A_{i}$ is the visual attention mask generated from the mask prediction of the previous round. $M_{T}$ denotes the text padding mask. Accordingly, the dual-path cross-attention can be expressed as follows:

(12) $$\tilde{Q}=LN\left[Q+{MHA}_{vis}\left(Q+Q_{pos},V_{i}^{\prime }+E_{i},V_{i}^{\prime };A_{i}\right)+{MHA}_{text}\left(Q+Q_{pos},T_{word}+E_{T},T_{word};M_{T}\right)\right]$$

At each Transformer decoder layer, the object queries perform cross-modal refinement by attending to both the language-enhanced visual memory and the word-level textual memory. The visual branch retrieves spatial object evidence from the current-scale visual memory modulated by LGC Gate, while the textual branch extracts fine-grained semantic constraints from the word-level textual memory. The two modality-specific representations are then integrated through dual-branch cross-attention to update the query representation $\tilde{Q}$. The updated query representation is further refined by query self-attention to capture interactions among different object queries, followed by a feed-forward network for query-wise nonlinear transformation.

(13) $$\begin{array}{cc} Q_{SA} & =LN\left(\tilde{Q}+{MHA}_{self}\left(\tilde{Q}+Q_{pos},\tilde{Q}+Q_{pos},\tilde{Q}\right)\right),\\ Q_{next} & =LN\left(Q_{SA}+FFN(Q_{SA})\right) \end{array}$$

Here, $\tilde{Q}$ denotes query representation, and $Q_{pos}$ represents the query positional embedding.

The Transformer mask decoder consists of $N_{d}$ serially stacked decoding layers. Each layer executes sequentially in the order of the LGC Gate, dual-path vision–language cross-attention, query self-attention, and feed-forward network. It then passes $Q_{\mathrm{next}}$ as the query input to the subsequent layer. The layers cyclically select the current-scale visual memory in the order of $P_{2}$, $P_{3}$, and $P_{4}$. The shared decoder sentence-level textual features are used for the LGC Gate in each layer. The word-level textual features are used for the textual cross-attention. Both of these textual features, along with the query positional encodings, are reused across all decoding rounds. In the implementation, each decoding layer possesses independent parameters for the LGC, cross-attention, self-attention, and FFN modules. In contrast, the elements passed and updated layer by layer are the query representations and their generated visual attention masks.

After completing each round of query updates, the decoder applies LayerNorm to the query representation $Q_{\mathrm{next}}$. It then generates category logits and mask embeddings through two shared prediction heads, respectively. Let $E_{\mathrm{mask}}^{q}$ denote the mask embedding corresponding to the q-th query. Let $F_{\mathrm{mask}}$ represent the high-resolution pixel-level features. The mask logit for this query at position p is calculated as follows:

(14) $$M_{pred}^{q}(p)=\left\langle E_{mask}^{q},F_{mask}(p)\right\rangle$$

Therefore, each query generates one candidate mask. All queries collectively form Q candidate mask maps. Intuitively, after multiple rounds of vision–language interactions, the queries form target-relevant mask embeddings. Meanwhile, the pixel-level features provide a local visual description for each spatial position. The dot product of these two elements measures the degree of matching between the current position and the target represented by the query. A larger dot product result indicates a higher probability that the position belongs to the target region corresponding to that query.

Let $c_{q}$ denote the foreground category logit of the q-th query in the final layer. Let $m_{q}$ represent its corresponding mask logit map. The model selects the query with the highest foreground response as the final output query:

(15) $$q^{*}=\arg\underset{q}{\max}c_{q}$$

Subsequently, the model extracts $m_{q^{*}}$, crops its padded regions, and interpolates it back to the original image size. Let this post-processing operation be denoted as R(·). The final segmentation mask is expressed as follows:

(16) $$M_{\mathrm{final}}=I\left[R(m_{q^{*}})>0\right]$$

Here, I[·] denotes the indicator function. The implementation utilizes a mask logit of 0 as the threshold. This is mathematically equivalent to applying a Sigmoid activation and subsequently using 0.5 as the threshold to generate the final foreground mask. The candidate masks generated by the initial queries and the intermediate decoding layers are solely used to construct the subsequent visual attention masks and for deep supervision. Only the mask corresponding to the query with the highest category score in the final layer serves as the final segmentation result.

#### 3.1.5. Loss Function

To equip the network with both reliable referring region segmentation capabilities and expression-relevant dynamic query initialization, we employ a joint optimization objective. This objective consists of a mask set prediction loss and SPLM auxiliary constraints. The former directly supervises the category and mask outputs of the decoder. The latter acts solely on the spatial prior branch prior to dynamic query selection. This serves to increase the probability that candidate locations fall within the referring target regions. Let the final total loss be defined as:

(17) $$L=L_{seg}+\lambda_{loc}\alpha (t)L_{loc}+\lambda_{rank}^{P2}L_{rank}^{P2}+\lambda_{rank}^{P3}L_{rank}^{P3}$$

Here, $L_{\mathrm{seg}}$ is the mask set prediction loss. $L_{\mathrm{loc}}$ is the SPLM spatial localization loss. $L_{\mathrm{rank}}^{P2}$ and $L_{\mathrm{rank}}^{P3}$ constrain the ranking of the prior maps at the P2 and P3 scales, respectively. In the final configuration, we set $\lambda_{\mathrm{loc}}=1.0$ and $\lambda_{\mathrm{rank}}^{P2}=\lambda_{\mathrm{rank}}^{P3}=0.1$.

(1) Mask Set Prediction Loss.

Given $N_{q}=50$ object queries, the model first correlates the prediction set with the ground-truth referring targets one-to-one via Hungarian matching. The matching cost is jointly composed of a category term, a mask binary cross-entropy term, and a Dice term. Their weights are set to 2.0, 5.0, and 5.0, respectively. For any decoding layer d, let the matched category predictions, mask logits, and binary ground-truth masks be denoted as $C^{\left(d\right)}$, $Z^{\left(d\right)}$, and Y, respectively. The supervision term for this layer is formulated as:

(18) $$L_{seg}^{\left(d\right)}=2L_{cls}^{\left(d\right)}+5L_{bce}^{\left(d\right)}+5L_{dice}^{\left(d\right)}$$

Here, $L_{\mathrm{cls}}$ is the cross-entropy loss including a no-object category. The weight for the no-object category is set to 0.1. This reduces the dominant effect of a large number of unmatched queries during training. For mask supervision, we adopt an uncertainty-guided point sampling strategy. Specifically, we select 12,544 sampling points from regions with high prediction uncertainty. This process utilizes an oversampling rate of 3.0 and an importance sampling ratio of 0.75. Let Ω denote the set of sampled points. The binary cross-entropy loss is expressed as:

(19) $$L_{bce}=-\frac{1}{\left|\Omega \right|}\sum_{u\in \Omega }\left[Y_{u}\log\sigma (Z_{u})+(1-Y_{u})\log(1-\sigma (Z_{u}))\right]$$

Here, σ(·) represents the Sigmoid function. The Dice loss is defined as:

(20) $$L_{dice}=1-\frac{2\sum_{u\in \Omega }\sigma (Z_{u})Y_{u}+1}{\sum_{u\in \Omega }\sigma (Z_{u})+\sum_{u\in \Omega }Y_{u}+1}$$

To alleviate the optimization difficulties of the deep decoder, this paper applies the aforementioned classification, BCE, and Dice supervision to the final prediction as well as the outputs of all intermediate decoding layers. Specifically,

(21) $$L_{seg}={\sum_{d\in D}L_{seg}}^{\left(d\right)}$$

Here, D represents the set containing the final decoding layer and all intermediate layers that actually generate predictions. The intermediate layer predictions are solely used for deep supervision. They do not participate in the mask output during the final inference stage.

(2) SPLM Spatial Localization Loss.

The goal of the SPLM is not to directly generate the final segmentation mask. Instead, it provides a target-directed spatial prior before dynamic query initialization. Therefore, we apply a lightweight localization constraint to the refined prior maps that are actually used for the P2 and P3 candidate ranking. Let ${\hat{M}}_{s}$ denote the prior prediction map at the s-th scale. Here, s∈{P2,P3}. First, the ground-truth referring mask Y is downsampled to the corresponding feature scale. Then, its spatial centroid $\left(c_{x},c_{y}\right)$ is calculated. Next, a 2D Gaussian target map centered at this centroid is constructed:

(22) $$G_{s}(x,y)=\exp\left(-\frac{(x-c_{x}{)}^{2}+(y-c_{y}{)}^{2}}{2\delta^{2}}\right)$$

Here, δ=2.0 denotes the standard deviation of the Gaussian kernel. Its unit is the feature map grid. Subsequently, a Soft Dice loss is employed to constrain the consistency between the prior predictions and the target response regions:

(23) $$L_{loc}=\frac{1}{2}\sum_{s\in \{P2,P3\}}\left(1-\frac{2\langle {\hat{M}}_{s},G_{s}\rangle +1}{\|{\hat{M}}_{s}{\|}_{1}+\|G_{s}{\|}_{1}+1}\right)$$

During the early stages of training, the decoder features and the SPLM refinement branch are not yet stable. Considering this, the weight grows linearly from near zero to the full weight over the first 2000 iterations. The adjustment coefficient is defined as:

(24) $$\alpha (t)=\min\left(\frac{t+1}{2000},1\right)$$

(3) Scale-Specific Query Ranking Loss.

Relying solely on the localization loss may cause the prior map to generate responses near the target center. However, it cannot guarantee that the Top-K candidates actually selected by the dynamic queries are located within the referring region. To address this, we apply list-wise ranking constraints to the P2 and P3 candidate scales, respectively. Let $A_{s}$ denote the prior ranking logits at scale s. Let $V_{s}$ represent the set of valid feature locations. Let $P_{s}\subseteq V_{s}$ denote the set of positive locations obtained from the ground-truth mask via adaptive max pooling. The loss is defined as:

(25) $$L_{rank}^{s}=\log\sum_{u\in V_{s}}\exp\left(\frac{A_{s}(u)}{\tau }\right)-\log\sum_{u\in P_{s}}\exp\left(\frac{A_{s}(u)}{\tau }\right),\;s\in \{P2,P3\}$$

Here, the temperature parameter τ is set to 0.1. This objective is equivalent to maximizing the softmax probability mass of the positive locations among all valid locations. Therefore, it only requires at least one target-relevant location to obtain a high response. It does not force the entire target region to possess uniformly high scores. The ground-truth mask employs max pooling instead of area-average downsampling. This prevents tiny objects from being diluted or disappearing within the P2 and P3 feature grids.

It must be emphasized that the query ranking loss supervises the SPLM prior logits. It does not supervise the predicted masks of the decoder. Consequently, it is functionally complementary to $L_{\mathrm{seg}}$. The former optimizes the quality of the initialization candidates for the dynamic queries. The latter optimizes the final pixel-level segmentation results after cross-modal decoding. Together, they enable the model to first filter out highly target-relevant candidate locations from complex backgrounds. Subsequently, the model accomplishes region restoration and boundary modeling through the downstream Transformer decoder.

Here, t denotes the current training iteration number. This progressive strategy prevents early unstable priors from exerting excessive interference on the main segmentation task. Simultaneously, it allows the SPLM to gradually learn target-relevant high-response regions during training.

### 3.2. Materials and Experimental Setup

#### 3.2.1. Materials

To evaluate the effectiveness and generalization capability of the proposed model, extensive experiments are conducted on two publicly available and challenging remote sensing referring image segmentation benchmarks, namely RefSegRS and RRSIS-D.

(1) RefSegRS Dataset

RefSegRS is a public benchmark constructed for the remote sensing referring expression segmentation task. It contains a total of 4420 annotated “image-text-mask” triplets, covering 285 real remote sensing scenarios. In this paper, we strictly follow its official data split. The training set, validation set, and test set contain 2172, 431, and 1817 samples, respectively. These account for 49.14%, 9.75%, and 41.11% of all samples. This dataset covers 14 typical geospatial object categories, including trucks, trailers, buses, bikeways, and trees. It also provides attribute labels to describe the categories, appearances, and spatial relationships of the targets.

The original size of the RefSegRS images is uniformly 512 × 512 pixels. Its maximum spatial resolution reaches 0.13 m. This places high demands on the model’s capabilities regarding local detail modeling and boundary restoration. In this paper, we adopt 512 pixels as the scale benchmark for aspect-ratio-preserving scaling during the training phase. We do not forcibly resample arbitrary images to a fixed size. This strategy avoids introducing additional magnification to the original images. Consequently, it reduces the negative impact of repeated interpolation on small objects, elongated structures, and local boundary textures. According to the unified preprocessing protocol detailed in Section 3.2.3, the images are directly fed into the network after the aspect-ratio-preserving scaling. We do not perform additional fixed-size random cropping. This prevents the referring targets from being truncated by the cropping operations. This preprocessing setting remains consistent across all comparison models and ablation variants trained in this study.

Only the official training set is utilized during the training phase. To ensure consistent experimental comparison criteria, the number of training iterations, learning rate scheduling, and other hyperparameters are fixed prior to the experiments. We do not perform model selection based on the metrics from the validation or test sets. Prior to evaluation, we check the size consistency between the images and their corresponding masks. All RefSegRS test results are reported on the complete official test set containing 1817 samples.

(2) RRSIS-D Dataset

Compared to RefSegRS, RRSIS-D is a larger remote sensing referring image segmentation benchmark dataset featuring greater scene diversity. It contains a total of 17,402 annotated “image-text-mask” triplets. We strictly follow the standard split of the RRSIS-D public benchmark in this paper. The training set, validation set, and test set contain 12,181, 1740, and 3481 samples, respectively. These account for 70.00%, 10.00%, and 20.00% of all samples, respectively. This dataset covers 20 main categories of geospatial objects. These include airplanes, vehicles, buildings, golf fields, and stadiums.

The image dimensions in RRSIS-D are uniformly 800 × 800 pixels. The original spatial resolution spans a wide range from approximately 0.5 m to 30 m. Significant target scale variations across different scenes, complex background interference, and appearance similarities among categories pose substantial challenges. They increase the difficulty of cross-scale feature fusion, expression-relevant object localization, and boundary restoration. Therefore, this dataset effectively evaluates the model’s adaptability in complex remote sensing scenarios.

Prior to data loading and evaluation, we perform consistency checks between the image sizes and their corresponding mask sizes across all data splits. We discovered that the image sizes of four samples in the official test set and two samples in the validation set do not match their annotations. Reliable inference and IoU calculations cannot be performed on these samples. Therefore, our proposed method and the corresponding ablation variants adopt the identical filtering rule across all random seeds. We report the metrics on 3477 valid test samples and 1738 valid validation samples, respectively. Unless otherwise specified, the main results for RRSIS-D are evaluated on the test set. The structural ablations, dynamic query quota analysis, and candidate localization diagnostics are conducted on the validation set.

RefSegRS and RRSIS-D were selected because they provide public RRSIS benchmarks with official splits, established metrics, and published baselines. RefSegRS emphasizes high-resolution scenes with relatively continuous targets, whereas RRSIS-D introduces greater scene diversity, wider scale variation, and stronger background interference. Together, they provide complementary evaluation conditions.

#### 3.2.2. Metrics

To quantitatively evaluate the performance of the proposed model on the remote sensing referring image segmentation task, this paper adopts three widely used evaluation metrics, including mean Intersection over Union (mIoU), overall Intersection over Union (oIoU), and Precision at IoU threshold X (Pr@X), providing a comprehensive assessment from multiple perspectives.

Mean Intersection over Union (mIoU) measures the average segmentation accuracy across all samples in the current evaluation set. Specifically, it first calculates the Intersection over Union between the predicted mask and the ground-truth annotation mask for each sample. Then, it computes the arithmetic mean of the IoUs across all samples. This metric reflects the sample-level segmentation quality. It prevents large-area targets from completely dominating the statistical results. The formulation is defined as follows:

(26) $$mIoU=\frac{1}{N}\sum_{i=1}^{N}\frac{\left|{\hat{Y}}_{i}\cap Y_{i}\right|}{\left|{\hat{Y}}_{i}\cup Y_{i}\right|}$$

where N denotes the total number of test samples, and ${\hat{Y}}_{i}$ and $Y_{i}$ represent the predicted mask and ground-truth mask of the i-th sample, respectively.

Overall Intersection over Union (oIoU) is computed by dividing the total number of intersection pixels by the total number of union pixels across all test samples. Compared with mIoU, oIoU assigns larger weights to large objects and emphasizes the completeness and consistency of large-scale object segmentation. The formulation is defined as follows:

(27) $$\mathrm{oIoU}=\frac{\sum_{i=1}^{N}|{\hat{Y}}_{i}\cap Y_{i}|}{\sum_{i=1}^{N}|{\hat{Y}}_{i}\cup Y_{i}|}$$

Precision at IoU threshold X (Pr@X) measures the proportion of test samples whose predicted IoU values exceed a predefined threshold X. This metric evaluates the localization accuracy and segmentation reliability of the model under different levels of evaluation strictness. The formulation is defined as follows:

(28) $$\mathrm{Pr}@X=\frac{1}{N}\sum_{i=1}^{N}I(\mathrm{IoU}\geq X)$$

where I(·) denotes the indicator function. In this paper, the model performance is evaluated under five thresholds, namely Pr@0.5, Pr@0.6, Pr@0.7, Pr@0.8, and Pr@0.9, to characterize its robustness in accurately locating fine-grained object boundaries.

#### 3.2.3. Experimental Setup

During the data preprocessing stage, the training images are first adjusted according to an aspect-ratio-preserving scaling strategy. The short edge of each image is scaled to 512 pixels, while the long edge is restricted to no more than 1024 pixels. According to the data processing code, no additional fixed-size random cropping is performed during training. The scaled images are directly fed into the network. To enhance the model’s adaptability to imaging differences and orientation changes in remote sensing images, random brightness, contrast, and saturation perturbations are applied during training. Additionally, random rotation is performed within an angle range of [−15°, 15°]. Random horizontal flipping is disabled to avoid altering the direction-sensitive spatial semantics in remote sensing scenes. During testing, only the same aspect-ratio-preserving scaling is applied, and no random data augmentation is used.

The entire network is implemented using the PyTorch deep learning framework (version 2.4.1). All experiments are conducted in a Linux environment on a single NVIDIA RTX 4090 GPU (NVIDIA, Santa Clara, CA, USA) with a batch size of 4. The hardware configuration includes a 13th Gen Intel Core i7-13700KF CPU (Intel, Santa Clara, CA, USA) and approximately 62 GB of system memory. The software stack relies on Python (version 3.12.13), accelerated by CUDA (version 11.8) and cuDNN (version 9.1.0). For the model architecture, InternImage-B is adopted as the visual backbone, and CLIP ViT-B/16 is used as the text encoder.

The model employs the AdamW optimizer with a weight decay coefficient of 0.05. Gradient clipping with a maximum norm of 0.01 is applied to the gradients of the entire model to improve numerical stability during training. The initial learning rate for the non-backbone network parameters is set to 5 × 10^−5^. The InternImage backbone uses a learning rate scaling factor of 0.1, resulting in an initial learning rate of 5 × 10^−6^. The learning rate is scheduled using WarmupMultiStepLR. A brief warm-up is performed during the first 10 iterations, after which the learning rate is decayed by a factor of 0.1 at the 10,000th and 20,000th iterations, respectively. The model is trained for 30,000 iterations on RefSegRS and 44,000 iterations on RRSIS-D.

Under this hardware and software configuration, the proposed RS-CARES model has a total of 286.2 M parameters (with 136.6 M trainable parameters). Evaluated at a standard input resolution of 512 × 512, the estimated computational cost is approximately 312.9 GFLOPs.

## 4. Experimental Results and Discussion

### 4.1. Comparison Results on the RefSegRS Dataset

As shown in Table 1, our proposed method achieves the optimal results across all five Pr metrics on the RefSegRS test set. The values for Pr@0.5, Pr@0.6, Pr@0.7, Pr@0.8, and Pr@0.9 are 84.04%, 77.77%, 66.04%, 40.29%, and 9.74%, respectively. The mIoU reaches 70.43%. This represents an improvement of 2.66 percentage points over FIANet. Notably, it achieves a significant increase of 10.10 percentage points under the Pr@0.8 metric. This indicates that the model maintains strong segmentation stability even under strict region overlap conditions. Compared to LAVT, CrossVLT, LGCE, and RMSIN, the mIoU of our method increases by 12.69, 14.30, 10.35, and 10.67 percentage points, respectively. This demonstrates that CLIP semantic modeling, multi-scale feature fusion, and dynamic query initialization effectively improve target localization and category discrimination in complex remote sensing scenarios.

The oIoU of our method is 77.34%. This is lower than the 78.90% achieved by FIANet. Furthermore, the Pr@0.9 is only 9.74%. This suggests that while the model performs well in terms of sample-average performance and category discrimination, insufficient region coverage and boundary deviations still exist for some targets. This limitation is particularly evident for large-scale targets, elongated structures, and targets with complex contours. Future improvements can be explored from the perspectives of high-resolution mask prediction, region integrity constraints, and boundary refinement.

As shown in Table 2, the average IoU of our proposed method across the 14 target categories on the RefSegRS test set reaches 66.20%. This is higher than the 65.85% achieved by FIANet. It improves upon LGCE and RMSIN by 10.98 and 11.35 percentage points, respectively.

Our method achieves the optimal results on 8 categories, including vehicle, car, van, building, truck, bus, road marking, and bikeway. Among them, the IoUs for vehicle, car, van, and truck reach 74.29%, 72.53%, 66.42%, and 79.50%, respectively. These represent improvements of 5.06, 4.88, 6.16, and 6.00 percentage points over FIANet, respectively. This indicates that the model possesses strong semantic discrimination and regional localization capabilities in scenarios featuring similar appearances and dense target distributions, such as vehicles and their sub-categories. For road marking, our method achieves 25.04%. This is an increase of 2.49 percentage points compared to FIANet. This demonstrates that cross-modal semantic modeling and high-resolution query initialization can improve the recognition performance for elongated targets characterized by low area proportions.

For categories including road, trailer, sidewalk, tree, low vegetation, and impervious surface, our method has not yet achieved the optimal results. Notably, the IoU for the tree category is 70.76%. This is lower than the 82.65% achieved by FIANet and represents the most obvious performance shortcoming at present. The results for road, trailer, and sidewalk also trail behind FIANet by 1.54, 3.00, and 1.88 percentage points, respectively. These categories typically possess large spatial coverage areas, complex texture variations, or irregular boundaries. Relying solely on query initialization and semantic matching makes it difficult to fully resolve issues regarding region integrity and boundary adherence. Therefore, future work still needs to strengthen the holistic region modeling of large-scale targets, the discrimination of vegetation textures, and the high-resolution refinement of complex boundaries.

### 4.2. Comparison Results on the RRSIS-D Dataset

Compared to RefSegRS, RRSIS-D possesses a larger data scale, richer geospatial categories, and more significant spatial resolution variations. This places higher demands on the model’s capabilities regarding object localization, scale adaptation, and boundary delineation. Table 3 adopts the official RRSIS-D test set split. It presents an overall performance comparison of different methods on this test set.

As shown in Table 3, RRSIS-D possesses a larger data scale, richer target categories, and more obvious spatial resolution variations. Therefore, it places higher demands on the model’s capabilities regarding cross-scale feature fusion, semantic understanding of complex scenes, and small object localization. On the RRSIS-D test set, the Pr@0.5, Pr@0.6, Pr@0.7, Pr@0.8, and Pr@0.9 of our proposed method reach 73.48%, 66.26%, 54.96%, 38.94%, and 21.02%, respectively. The oIoU and mIoU reach 73.68% and 63.71%, respectively. This final result is the average of independent training results obtained using three different random seeds. Notably, our method achieves the highest mIoU in the table. It improves upon FIANet, RMSIN, LGCE, and CrossVLT by 1.28, 2.06, 3.01, and 3.32 percentage points, respectively. This demonstrates that the model possesses a higher average segmentation quality on the RRSIS-D evaluation samples. Combined with the category-level results in Table 4, our method achieves leading results on multiple categories. These include airplanes, baseball fields, golf fields, chimneys, bridges, and windmills. This indicates that it possesses a certain degree of cross-category generalization ability.

Regarding the Pr metrics, our method reaches 73.48% on Pr@0.5. This is slightly higher than the 73.11% achieved by FIANet, representing an increase of 0.37 percentage points. This indicates that the model can stably locate the predicted region near the target. However, as the IoU threshold increases, the gap between our method and FIANet gradually widens. Our method falls behind FIANet by 0.44, 1.49, 2.63, and 2.77 percentage points on Pr@0.6, Pr@0.7, Pr@0.8, and Pr@0.9, respectively. This suggests that the current model possesses advantages in rough target localization and sample-average segmentation quality. However, shortcomings still exist regarding strict region overlap, fine-grained boundary adherence, and the complete coverage of complex targets. Specifically, the Pr@0.9 is only 21.02%. This illustrates that while some samples can be correctly localized, boundary deviations, local omissions, or region expansions still exist between the predicted masks and the ground-truth masks.

It is worth noting that the mIoU of our method is higher than that of FIANet. However, the oIoU is 73.68%. This is lower than FIANet’s 75.88%, resulting in a difference of 2.20 percentage points. This discrepancy indicates that our method can achieve a higher segmentation quality in a sample-average sense. However, when all pixels are uniformly aggregated, regional coverage errors for some large-area targets or high-frequency categories still significantly impact the overall Intersection over Union. In other words, the primary advantages of the current model are reflected in cross-modal semantic understanding, target category discrimination, and candidate region localization. Conversely, there remains room for further improvement in the overall coverage capabilities for large-scale structures like ports, airports, and roads, as well as complex boundaries. Future improvements can focus on high-resolution mask decoding, region integrity constraints, and boundary refinement supervision. This will help narrow the gap between mIoU and oIoU and improve the strict matching capabilities under high IoU thresholds.

Overall, our proposed method achieves the highest mIoU on the RRSIS-D test set. It also achieves optimal performance on the low-threshold localization metric Pr@0.5. This demonstrates that the proposed cross-modal context aggregation, semantic prior localization, and dynamic query initialization can effectively enhance the model’s capabilities in recognizing and segmenting complex remote sensing targets. Simultaneously, the lower high-threshold Pr and oIoU scores reveal the current method’s limitations in precise boundary restoration and the complete segmentation of large regions. This provides a clear direction for subsequent model improvements.

Combined with the category-level results in Table 4, our proposed method achieves leading results on the categories of baseball fields, golf fields, chimneys, bridges, airplanes, and windmills. Among them, the average IoUs for the baseball field and golf field categories are 83.37% and 76.38%, respectively. These represent improvements of 14.99 and 20.78 percentage points over FIANet, respectively. The categories of chimneys, bridges, airplanes, and windmills show improvements of 10.51, 15.12, 8.65, and 16.52 percentage points, respectively. The aforementioned categories typically possess relatively distinguishable regional layouts, main structures, or texture differences. This indicates that the proposed spatial prior and cross-scale feature interactions can provide more effective candidate guidance for expression-relevant regions. Furthermore, they help maintain the complete coverage of the main target bodies.

It must still be noted that our method has not yet achieved optimal results on categories such as airports, harbors, expressway service areas, basketball courts, tennis courts, and dams. For example, the average IoUs for the airport and harbor categories are 53.41% and 31.74%, respectively. These are lower than the 67.20% and 60.18% achieved by FIANet. These categories often contain multi-component regions with large scale spans, dense background textures, or boundaries similar to surrounding geospatial objects. These factors easily cause insufficient candidate region coverage and local boundary deviations. Future methods can focus on improving upon these specific issues.

### 4.3. Qualitative Results

#### 4.3.1. Final Mask Quality on RefSegRS

**Figure 7:**
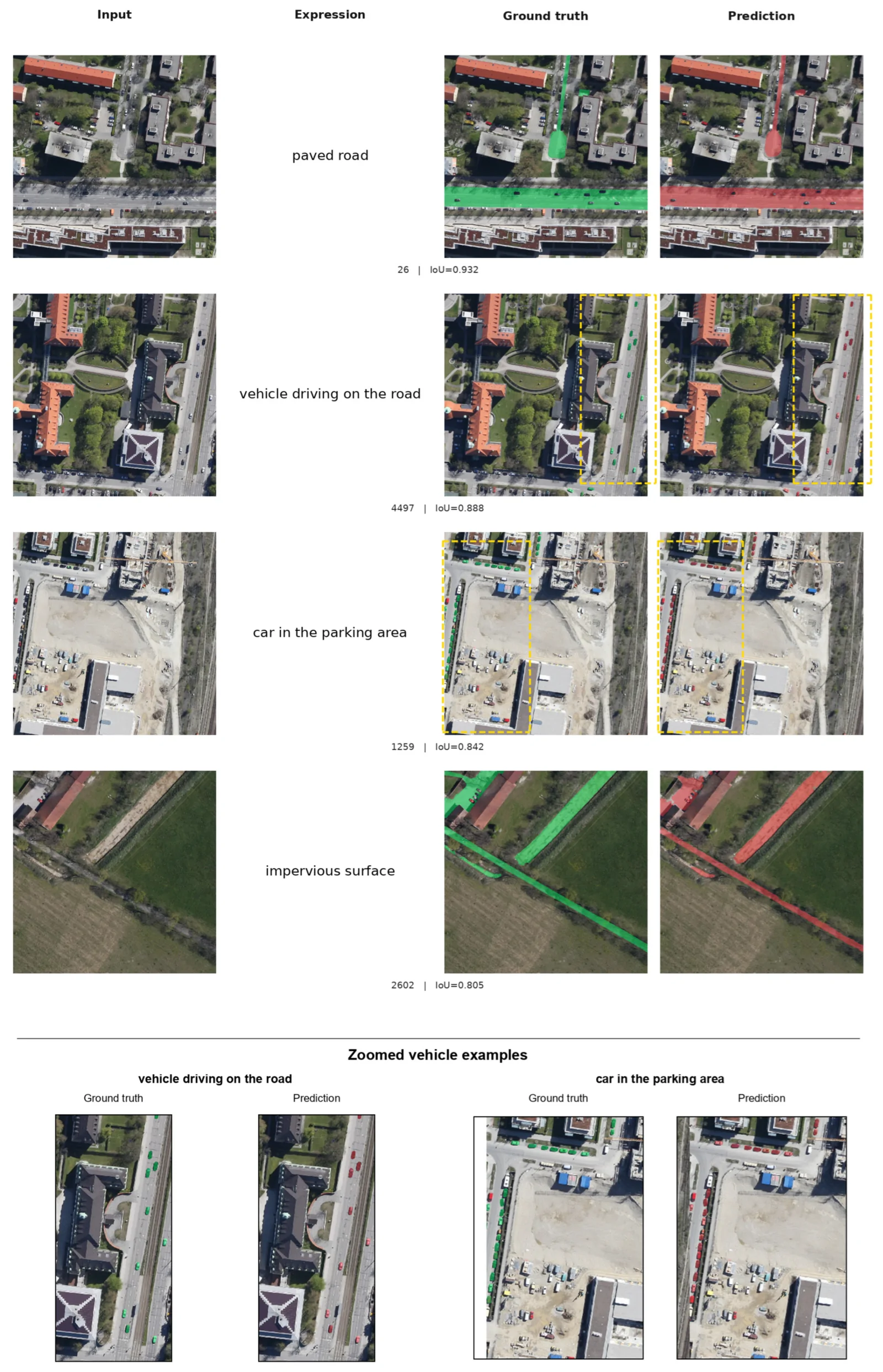
Visualization results on the RefSegRS dataset. Zoomed views are provided below for the two vehicle examples.

As shown in Figure 7, the columns from left to right display the input image, referring expression, ground truth, and model prediction results, respectively. The IoU for the corresponding sample is provided below each prediction result. The figure selects four distinct scenes. These include paved roads, vehicles driving on the road, cars in the parking area, and impervious surfaces. This selection aims to cover large-area continuous targets, discrete small targets, and targets with complex boundaries. The IoUs for the four examples are 0.932, 0.888, 0.842, and 0.805, respectively.

For the “paved road”, the model can completely aggregate the continuous road regions. It also maintains good boundary consistency. For the “vehicle driving on the road” and “car in the parking area”, the model successfully filters out expression-relevant vehicle targets from complex backgrounds such as roads or parking lots. This demonstrates its capability in small target localization. However, slight under-segmentation still exists at the edges of some vehicles. For the “impervious surface”, the prediction results successfully cover the main hardened surface areas. Nevertheless, certain deviations remain at the narrow branches and the boundaries adjacent to grass and bare land.

Overall, RS-CARES successfully accomplishes the semantic localization and regional segmentation of targets across various scales and shapes based on the linguistic expressions. The predicted masks demonstrate high consistency with the ground-truth annotations in the main body regions. This phenomenon aligns with the quantitative results obtained on the RefSegRS dataset. The model performs stably on targets possessing clear structures, such as buildings, impervious surfaces, and vehicles. Conversely, there remains room for further improvement regarding the boundary delineation of elongated structures, low-texture regions, and small-scale targets.

#### 4.3.2. Final Segmentation Results and Error Distribution on RRSIS-D

**Figure 8:**
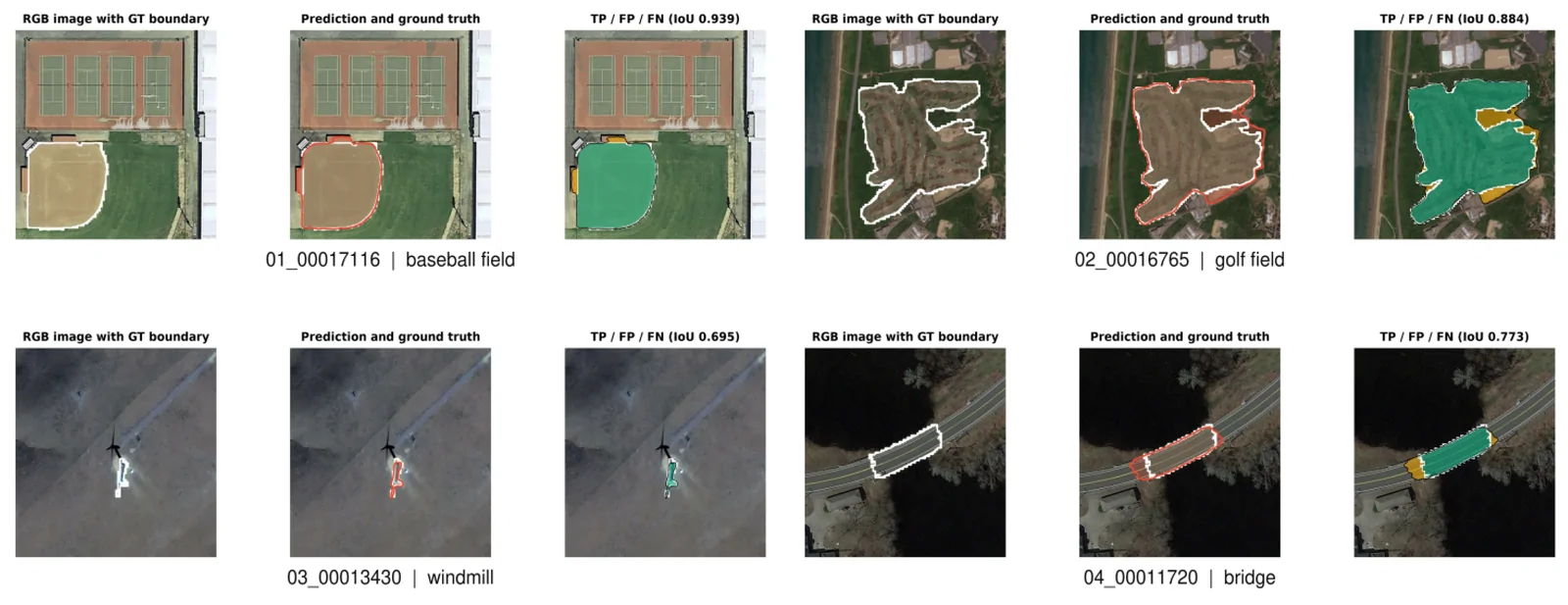
Final Segmentation Results and Error Distribution of Representative Examples on RRSIS-D.

Figure 8 selects four representative expressions: baseball field, golf field, windmill, and bridge. It presents the input images overlaid with the ground-truth and predicted boundaries. Additionally, it provides the corresponding TP/FP/FN error maps. The IoUs for the four examples are 0.939, 0.884, 0.695, and 0.773, respectively. These examples cover large-area regular targets, targets with complex contours, extremely small targets, and elongated targets.

The results show that the model can restore the main bodies of the targets in most examples. It successfully restricts the primary errors to the contour edges. However, the windmill example still exhibits local missed detections. Furthermore, the bridge example shows minor deviations along the edges of its elongated structure. This set of figures serves to illustrate the region integrity and error morphology of the final masks. It is not intended to replace the overall metrics with individual examples.

#### 4.3.3. Multi-Scale Candidates and Spatial Hits of Dynamic Queries

**Figure 9:**
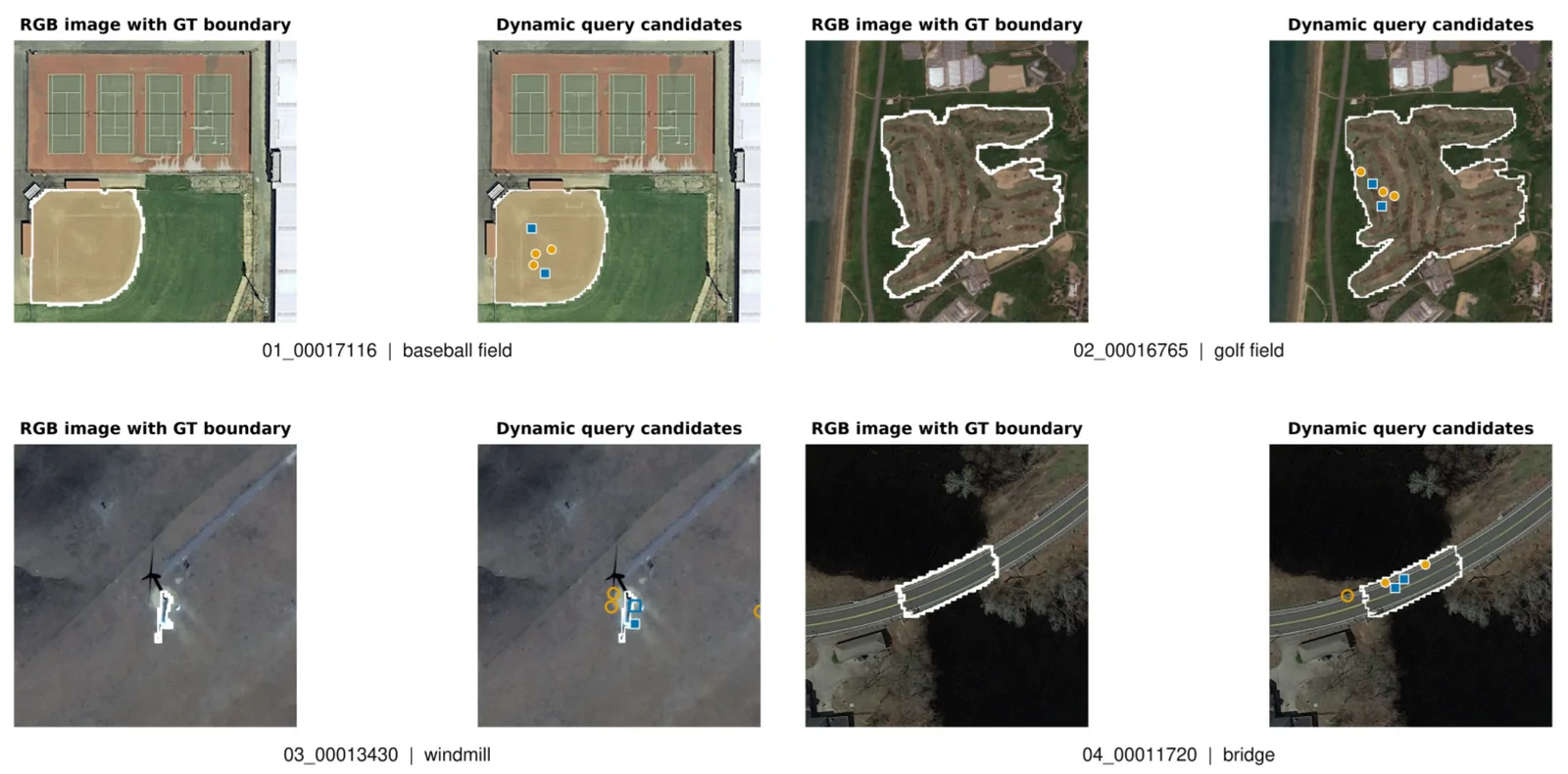
Candidate Locations and Target Hits of Dynamic Queries for Representative Examples on RRSIS-D.

Figure 9 further illustrates the spatial initialization process of the dynamic queries. Orange dots represent the P2 candidates. Blue squares represent the P3 candidates. Solid and hollow shapes indicate whether the candidate points are located inside or outside the ground-truth targets, respectively.

In the baseball field and golf field examples, candidates from both scales fall within the target regions. This demonstrates that fine-grained local evidence and larger receptive field contexts can complement each other. For the windmill example, the P2 candidates fail to hit the target. However, the P3 candidates still retain one candidate inside the target. This indicates that the coarser-scale branch can provide supplementation in extremely small target scenarios. In the bridge example, all P3 candidates successfully hit the target. Simultaneously, the P2 candidates provide local positions closer to the elongated boundaries.

This visualization supports the design that cross-layer quotas and spatial deduplication jointly improve candidate coverage. Nevertheless, it also reveals that extremely small targets remain the primary difficulty for candidate initialization.

#### 4.3.4. SPLM Semantic Seeds and Scale-Specific Refinement

**Figure 10:**
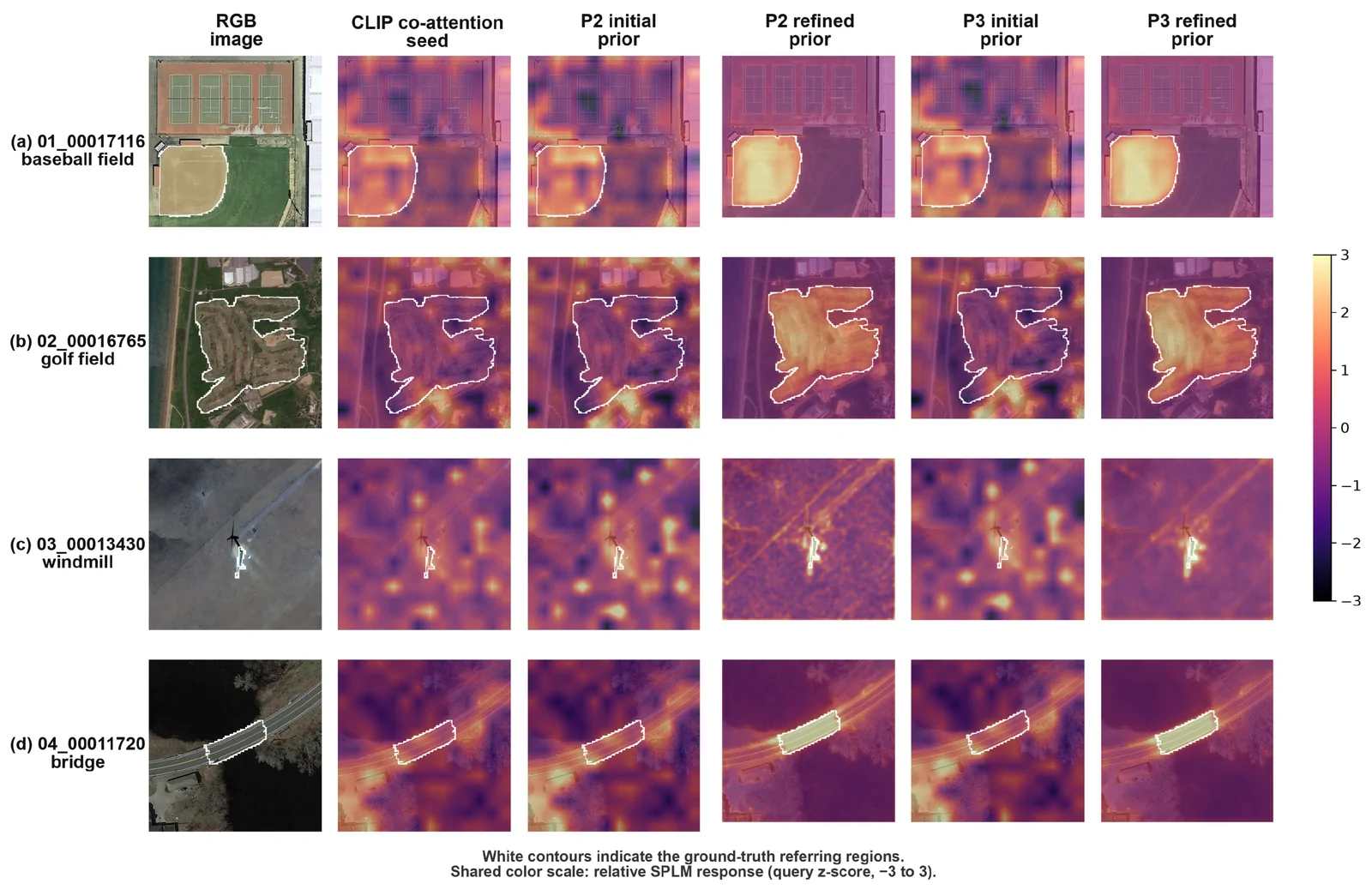
SPLM Semantic Seeds and Scale-Specific Prior Refinement of Representative Examples on RRSIS-D.

Figure 10 illustrates the spatial response variations of the same expression. These are displayed in the order of the RGB image, CLIP semantic seeds, P2/P3 initial priors, and refined priors.

The CLIP semantic seeds first provide coarse-grained responses related to the text. The P2 initial priors introduce high-resolution visual details while preserving the semantic responses. Subsequently, the P2 refined priors further enhance the responses within the target and suppress the background. The response range of the P3 branch is relatively smoother. It primarily undertakes the confirmation of the target region under a larger receptive field.

In the windmill example, the target response remains visible after refinement. However, the background texture interference is not yet completely eliminated. This indicates that the prior maps are primarily utilized for candidate ranking and initialization. They are not intended to directly replace the final mask prediction. Overall, Figure 10 illustrates the complementary relationship between the frozen CLIP semantic information and the scale-specific visual refinement from the perspective of spatial responses.

#### 4.3.5. Comprehensive Interpretation of Visual Evidence

The three sets of figures above correspond to three distinct levels. These include the final masks, candidate queries, and SPLM priors. The final mask figures reflect the regional reconstruction results of the decoder. The query figures indicate whether the candidates cover the targets before the decoding process begins. The SPLM figures explain the semantic spatial signals relied upon for candidate ranking.

These three components cannot replace one another. A high prior response does not imply that the pixel-level boundaries are already accurate. Similarly, a candidate hit does not guarantee that the final mask will completely cover the target. The qualitative results can be combined with the overall metrics and category statistics in Table 3 and Table 4. This combination provides a more comprehensive explanation of the effectiveness of RS-CARES in target localization, scale complementarity, and region refinement. It also highlights the remaining room for improvement regarding extremely small targets and low-contrast boundaries.

### 4.4. Ablation Studies and Component Contribution Analysis

#### 4.4.1. Ablation Study on Main Structures on the RRSIS-D Dataset

Table 5 indicates that under a unified training budget and the same foundational framework, the complete RS-CARES method achieves an mIoU of 64.30% and an oIoU of 74.39% on the RRSIS-D validation set. Compared to the B0 baseline, which only retains the static sentence-level language-conditioned query, the mIoU and oIoU of F0 improve by 15.09 and 11.89 percentage points, respectively. This demonstrates that the introduced cross-modal context, spatial priors, dynamic query, and decoder language interactions provide significant overall gains within this unified framework.

Judging from the results of the item-by-item removal, the mIoU drops to 51.74% (a decrease of 12.56 percentage points) after removing CCA. This indicates that multi-scale cross-modal context aggregation is one of the primary sources of performance improvement. After removing SPLM, the dynamic queries, and decoder language interactions, the mIoU decreases by 5.76, 4.44, and 5.84 percentage points, respectively.

The above results support several core design motivations. First, SPLM provides expression-relevant spatial priors for candidate regions. Second, the dynamic query transforms these priors into query initializations. Finally, LGC and word-level text cross-attention continuously inject language conditions during the layer-by-layer decoding process.

It should be noted that E2 jointly disables BAT, MSDeformAttn, and the language-guided BiFPN within CCA. Similarly, E5 jointly disables LGC and the text cross-attention. Therefore, these rows validate the overall effect of the corresponding module groups. They do not represent the independent causal contributions of each individual sub-component.

#### 4.4.2. Ablation Study on Main Structures on the RefSegRS Dataset

Table 6 shows that the B0 static query baseline achieves an mIoU of 66.06% and an oIoU of 75.85% on RefSegRS. This indicates that InternImage, the frozen CLIP text encoder, the standard multi-scale decoder, and Mask2Former already constitute a strong foundational framework. Building upon this, F0 improves the mIoU and oIoU to 70.43% and 77.34%, respectively. Therefore, the performance improvement cannot be attributed to a weak baseline.

Among all the modules, the role of CCA is the most explicit. After removing CCA, the mIoU drops by 2.41 percentage points, and the oIoU drops by 0.89 percentage points. This indicates that bidirectional vision–language interaction and multi-scale context modeling have a stable contribution to RefSegRS.

In contrast, the removal of SPLM and Dynamic Query does not cause a significant decline. The mIoUs of E3 and E4 are 70.47% and 70.65%, respectively. These are basically comparable to F0. Furthermore, E5 achieves the highest mIoU of 70.97% and oIoU of 77.97% after removing the language-guided decoding.

This result suggests that the targets in RefSegRS are typically large and regionally continuous. The basic decoder is already capable of accomplishing sufficient localization. The SPLM and Dynamic Query are specifically designed for small targets and complex backgrounds. Consequently, their marginal benefits on this dataset are limited. The layer-by-layer language guidance might even cause information redundancy with the cross-modal fusion during the encoding stage.

Consequently, F0 should be interpreted as a unified complete model adopted across multiple datasets. It is not necessarily the optimal model for every single metric on RefSegRS. Its module value needs to be further demonstrated in combination with the more complex small-target scenarios present in RRSIS-D.

#### 4.4.3. Ablation Study on Dynamic Query Scale Quotas

To verify the complementary effects of candidates from different scales in the dynamic query initialization, we compare three quotas: P2/P3 = 2/3, 3/2, and 4/1. Under these conditions, the total number of queries is fixed at 5.

P2 possesses a higher spatial resolution. It primarily provides local detail candidates. P3 possesses a larger effective receptive field. It primarily provides context-level candidates. The three sets of experiments adopt the identical RRSIS-D validation set, random seed, and training budget. They also utilize the same spatial non-maximum suppression and fusion scoring rules.

The three quotas are derived from independent 10k checkpoints. Therefore, this experiment is specifically utilized to analyze the scale behavior and the complementary structure of the candidates. It should not be interpreted as a strict comparison of fixed-weight optimality.

Table 7 adopts a unified definition of scale joint events. Let $H_{2}$ denote that the P2 branch has at least one candidate location falling within the ground-truth mask region within its quota $k_{2}$. Let $H_{3}$ denote that the P3 branch has at least one candidate location falling within the ground-truth mask region within its quota $k_{3}$. Based on this, we define Any-hit = $H_{2}\cup H_{3}$, Both = $H_{2}\cap H_{3}$, P2-only = $H_{2}⧵H_{3}$, and P3-only = $H_{3}⧵H_{2}$.

Although the 4/1 configuration achieves the highest Any-hit rate (86.94%), this result is primarily contributed by the P2 branch. The P2-only rate reaches 38.72%. Meanwhile, the P3-only rate is merely 1.61%. This indicates that a single P3 query struggles to form a stable and independent context compensation. In contrast, the P2-only and P3-only rates for the 3/2 configuration are 13.58% and 17.89%, respectively. The difference between the two is relatively small. This demonstrates that the query resources are not overly concentrated on a single scale.

Furthermore, the recovery rates are calculated using the same joint event system. The proportion of P2 missed samples recovered by P3 is calculated as P3-only/($1-H_{2}$). The proportion of P3 missed samples recovered by P2 is calculated as P2-only/($1-H_{3}$). Under the 3/2 configuration, the two recovery rates are 17.89%/(100–56.56%) = 41.19% and 13.58%/(100–60.87%) = 34.71%, respectively. This indicates that both scales can provide effective supplementation for targets not covered by the other branch. Conversely, the P3 recovery rate for the 4/1 configuration is only 10.98%. This reflects that the cross-scale compensation ability of P3 significantly weakens when its query quantity is insufficient.

Therefore, this paper adopts P2 = 3 and P3 = 2 as the default quotas. This decision is based on a more balanced scale contribution and bidirectional compensation. It does not claim to possess the highest Any-hit rate. The above statistics solely evaluate the candidate localization quality during the query initialization phase. They do not replace the final mask evaluation.

#### 4.4.4. SPLM-Guided Dynamic Query Candidate Localization Diagnosis

This paper aims to directly verify whether SPLM improves dynamic query initialization. It seeks to confirm that SPLM does not merely exert an indirect impact on the final mask prediction. Therefore, we compare the complete model F0 with the SPLM-removed model E3 on the RRSIS-D validation set.

Both models retain the identical InternImage backbone, CCA, dynamic query decoder, and language-guided decoding path. E3 removes the SPLM spatial priors, global visual prior vectors, P2/P3 prior refinement, and related auxiliary constraints. Furthermore, it adopts a candidate ranking mechanism relying solely on cosine similarity.

Let $H_{2}$ denote P2 Hit@3. Let $H_{3}$ denote P3 Hit@2. Consequently, we define Any-hit = $H_{2}\cup H_{3}$, P2-only = $H_{2}⧵H_{3}$, and P3-only = $H_{3}⧵H_{2}$. The intersection event, Both = $H_{2}\cap H_{3}$, can be calculated by $H_{2}+H_{3}$ − Any-hit.

Hit@K and its associated joint events are utilized to evaluate the candidate localization quality during the query initialization phase. The mIoU presented in the table is derived from the standard evaluation results of the corresponding checkpoints.

Table 8 indicates that after removing SPLM, the P2 Hit@3 and P3 Hit@2 of E3 are merely 3.34% and 2.59%, respectively. The Any-hit drops to 5.41%. The two branch hit rates of the complete F0 increase to 52.76% and 69.62%, respectively. The Any-hit reaches 77.62%.

In F0, P3-only is calculated as 77.62 − 52.76% = 24.86%. This indicates that the P3 branch can provide independent candidates for expressions missed by P2. The corresponding P3 recovery rate is 24.86%/(100 − 52.76%) = 52.62%. These results demonstrate the effectiveness of the SPLM-guided query initialization path. This path encompasses spatial priors, prior refinement, related auxiliary constraints, and global visual context injection. It can simultaneously improve the candidate localization quality of both P2 and P3 scales. It also enhances the compensation effect of cross-scale candidates.

To avoid inferring expression-wise changes from aggregated proportions, this paper further conducts a sample-wise paired statistical analysis. It compares the Any-hit for F0 and E3 based on the 1738 expressions in the same RRSIS-D validation set. The results show that 83 expressions are hit under both models. A total of 1266 expressions are hit by F0 but missed by E3. A total of 11 expressions are hit by E3 but missed by F0. A total of 378 expressions are missed under both models. The aforementioned 1266 and 11 represent expression-wise paired statistics. They are not directly deduced in reverse from the marginal proportions in Table 8. Their net difference of 1266 − 11 = 1255 is consistent with the overall improvement of Any-hit.

The improvement in the candidate hit rate occurs simultaneously with the improvement in final segmentation performance. In the standard validation results of the corresponding checkpoints, the mIoU of F0 is 64.30%. This is an increase of 5.76 percentage points compared to the 58.54% of E3.

Furthermore, in the scale joint events of F0, the P3-only candidate hit proportion is 24.86%. That is, the P3 branch can provide independent candidates for expressions missed by the P2 branch. Its recovery rate for expressions missed by P2 is 52.62%. This indicates that SPLM not only improves the target relevance of single-scale candidates. It also enhances the candidate compensation effect between the P2 and P3 scales.

This phenomenon is qualitatively consistent with the dual-scale balanced complementary characteristics demonstrated by the 3/2 quota in Table 7. It demonstrates that the current query allocation of P2 = 3 and P3 = 2 can balance high-resolution local detail candidates and context candidates. It must be emphasized that Hit@K evaluates the candidate coverage quality during the query initialization phase prior to decoding. The final mask still requires progressive optimization through subsequent vision–language cross-attention and mask prediction. Therefore, the candidate hit rate is not equivalent to the final segmentation quality.

### 4.5. Limitations and Practical Implications

RS-CARES improves expression-related candidate localization and sample-averaged segmentation quality in complex remote sensing scenes. The comparison and ablation results show that semantic spatial priors improve the selection of initial candidate locations, while Dynamic Query supports the complementary use of local details and contextual information. These properties are valuable for referring targets that are small, visually similar to surrounding objects, or described through spatial relations and attributes.

The results also reveal clear performance boundaries. On RRSIS-D, the proposed method achieves a higher mIoU than FIANet but a lower oIoU, and it does not obtain the best results at stricter IoU thresholds. Large-area objects, elongated structures, low-texture regions, and complex boundaries remain difficult. Predictions may contain incomplete regions, boundary offsets, or responses to similar neighboring objects. The model is therefore more suitable for expression-guided target localization and mask generation than for applications requiring highly precise boundary delineation over large regions.

The method provides a language-guided interface for remote sensing interpretation. Users can describe targets by category, attribute, location, scale, or contextual relation, then obtain candidate masks for roads, buildings, airports, ports, vehicles, and other geographic objects. This interaction is relevant to remote sensing segmentation and mapping workflows, including land-use and land-cover analysis and urban scene interpretation [1,34,35]. Model transfer may be affected by domain shifts across geographic environments and imaging conditions [36]. Future work will focus on improving large-region recovery and boundary consistency in diverse remote sensing scenes.

Future work will test RS-CARES on NWPU-Refer, RISORS, RIS-LAD, RSRefSeg 2, and SegEarth-R2 to evaluate its performance under their respective data distributions and annotation settings.

## 5. Conclusions

This work presents RS-CARES, a context-aware framework for referring remote sensing image segmentation. The model is built upon InternImage, a frozen CLIP text encoder, and the Mask2Former framework, and addresses expression-aware target localization and cross-modal feature aggregation in complex remote sensing scenes. Its main design consists of four coordinated components: (i) SPLM constructs expression-related spatial priors from frozen CLIP image and text representations and refines them with P2/P3 visual features for candidate ranking; (ii) CCA combines bidirectional attention transfer, multi-scale deformable attention, and a language-guided BiFPN to aggregate visual and textual context across scales; (iii) dynamic query initialization selects complementary P2/P3 candidates using scale allocation and spatial non-maximum suppression; and (iv) the language-guided decoder injects sentence-level context gating and word-level textual cross-attention during iterative mask decoding.

Experiments on RefSegRS and RRSIS-D validate the framework under a unified InternImage+CLIP+Mask2Former setting. On RRSIS-D, the complete F0 model achieves 64.30% mIoU and 74.39% oIoU on the validation set, improving over the static-query B0 baseline by 15.09 and 11.89 percentage points, respectively. The structural ablation results show that CCA, SPLM, dynamic query initialization, and the language-guided decoder jointly provide substantial gains in this challenging setting. In particular, the SPLM-guided query diagnostic demonstrates improved P2/P3 candidate coverage: compared with the SPLM-free model, F0 increases Any-hit from 5.41% to 77.62%, while the P2/P3 = 3/2 allocation provides a balanced cross-scale candidate complement. On RefSegRS, F0 also improves mIoU from 66.06% for the static-query baseline to 70.43%. However, the component-wise results indicate that the benefits of SPLM, dynamic query initialization, and decoder-level language guidance are data-dependent: they are most evident on RRSIS-D, where small targets, scale variation, and complex backgrounds create a stronger need for explicit spatial priors and cross-scale candidate selection, whereas RefSegRS contains more spatially continuous targets for which the base decoder already provides relatively strong localization.

## Figures and Tables

**Table 1:** Comparative experimental results on the RefSegRS test set.

Methods	Publication/Ref.	Pr@0.5	Pr@0.6	Pr@0.7	Pr@0.8	Pr@0.9	oIoU	mIoU
LSTM-CNN	ECCV 2016 [4]	15.69	10.57	5.17	1.10	0.28	53.83	24.76
ConvLSTM	CVPR 2018 [6]	31.21	23.39	15.30	7.59	1.10	66.12	43.34
CMSA	CVPR 2019 [24]	28.07	20.25	12.71	5.61	0.83	64.53	41.47
BRINet	CVPR 2020 [33]	22.56	15.74	9.85	3.52	0.50	60.16	32.87
LAVT	CVPR 2022 [7]	71.44	57.40	32.14	15.41	4.51	76.46	57.74
CrossVLT	TMM 2023 [8]	71.63	58.28	34.67	15.34	5.27	76.44	56.13
LGCE	TGRS 2024 [3]	75.34	67.37	44.78	18.21	5.03	76.71	60.08
RMSIN	CVPR 2024 [2]	71.78	55.89	31.58	10.77	1.53	76.49	59.76
FIANet	TGRS 2025 [5]	83.45	74.61	61.77	30.19	7.67	78.90	67.77
Ours		84.04	77.77	66.04	40.29	9.74	77.34	70.43

**Table 2:** Results of different categories on the RefSegRS test dataset.

Category	LGCE	RMSIN	FIANet	Ours
road	71.85	71.37	73.16	71.62
vehicle	61.20	60.79	69.23	74.29
car	59.00	58.60	67.65	72.53
van	40.38	40.11	60.26	66.42
building	79.58	79.05	80.27	80.87
truck	60.85	60.44	73.50	79.50
trailer	48.36	48.04	73.94	70.94
bus	44.03	43.74	71.45	72.04
road marking	6.46	6.42	22.55	25.04
bikeway	52.64	52.29	60.36	60.98
sidewalk	59.87	59.47	62.08	60.20
tree	65.69	65.25	82.65	70.76
low vegetation	42.40	42.12	44.25	42.55
impervious surface	80.73	80.19	80.46	78.99
average	55.22	54.85	65.85	66.20

**Table 3:** Experimental results of different image segmentation methods on the RRSIS-D test set.

Methods	Publication/Ref.	Pr@0.5	Pr@0.6	Pr@0.7	Pr@0.8	Pr@0.9	oIoU	mIoU
RRN	CVPR 2018 [6]	51.07	42.11	32.77	21.57	6.37	66.43	45.64
CMSA	CVPR 2019 [24]	55.32	46.45	37.43	25.39	8.15	69.39	48.54
LSCM	ECCV 2020 [25]	56.02	46.25	37.70	25.28	8.27	69.05	49.90
CMPC	CVPR 2020 [26]	55.83	47.40	36.94	25.45	9.19	69.22	49.24
BRINet	CVPR 2020 [33]	56.90	48.77	39.12	27.03	8.73	69.88	49.65
CMPC+	TPAMI 2021 [27]	57.65	47.51	36.97	24.33	7.78	68.64	50.24
LAVT	CVPR 2022 [7]	65.97	59.71	50.68	39.44	22.46	75.41	58.07
CrossVLT	TMM 2023 [8]	69.43	62.92	52.71	41.31	24.82	75.36	60.39
LGCE	TGRS 2024 [3]	68.96	62.36	52.74	40.09	23.13	75.17	60.70
RMSIN	CVPR 2024 [2]	70.83	64.40	54.43	42.00	24.28	75.39	61.65
FIANet	TGRS 2025 [5]	73.11	66.70	56.45	41.57	23.79	75.88	62.43
Ours	-	73.48	66.26	54.96	38.94	21.02	73.68	63.71

Note: The IoU for each category is calculated by dividing the cumulative intersection of all samples within that category by their cumulative union. The last row represents the arithmetic mean of the IoUs across all categories. This statistical approach differs from the sample-wise calculated mIoU.

**Table 4:** Results of different categories on the RRSIS-D test dataset.

Category	LGCE	RMSIN	FIANet	Ours
airplane	56.43	60.17	60.32	68.97
airport	67.33	67.15	67.20	53.41
golf field	55.71	55.30	55.60	76.38
expressway service area	76.36	75.67	76.20	65.90
baseball field	70.13	66.01	68.38	83.37
stadium	84.02	82.02	81.20	82.82
ground track field	81.68	80.85	78.20	77.13
storage tank	72.52	72.67	74.77	74.71
basketball court	73.55	71.29	73.50	66.72
chimney	68.06	67.49	66.40	76.91
tennis court	74.81	75.67	76.22	68.82
overpass	66.89	69.19	67.96	62.75
train station	57.46	61.80	59.47	61.67
ship	62.72	63.75	64.01	61.36
expressway toll station	60.88	64.81	62.90	71.19
dam	63.78	67.77	69.23	61.03
harbor	59.73	59.55	60.18	31.74
bridge	33.25	36.12	36.69	51.81
vehicle	42.48	46.90	48.12	49.07
windmill	40.13	41.32	45.25	61.77
average	63.40	64.29	64.60	65.38

Note: The IoU for each category is calculated by dividing the cumulative intersection of all samples within that category by their cumulative union. The last row presents the arithmetic mean of the IoUs across all categories. This statistical formulation differs from the mIoU, which is calculated on a per-sample basis.

**Table 5:** Ablation Study Results of Main Structures on the RRSIS-D Validation Set.

Setting	C	S	D	L	oIoU (%)	mIoU (%)
B0 Static Q	—	—	—	—	62.50	49.21
E2 w/o CCA	—	√	√	√	65.28	51.74
E3 w/o SPLM	√	—	√	√	68.41	58.54
E4 w/o DQ	√	√	—	√	71.62	59.86
E5 w/o L	√	√	√	—	70.56	58.46
F0 RS-CARES	√	√	√	√	74.39	64.30

Note: All configurations utilize the foundational frameworks of InternImage, CLIP, and Mask2Former. They are evaluated on the identical RRSIS-D validation set using the same random seed after 44,000 iterations. C, S, D, and L denote CCA, SPLM, Dynamic Query, and LGC+TCA, respectively. Specifically, CCA includes BAT, MSDeformAttn, and language-guided BiFPN. LGC+TCA encompasses the language-guided global context gating and the decoder word-level text cross-attention. F0 reports the average results across three random seeds. B0 retains the static sentence-level language-conditioned query. It should not be interpreted as a baseline without a language model.

**Table 6:** Ablation Study Results of Main Structures on the RefSegRS Test Set.

Setting	C	S	D	L	oIoU (%)	mIoU (%)
B0 Static Q	×	×	×	×	75.85	66.06
E2 w/o CCA	×	√	√	√	76.54	68.02
E3 w/o SPLM	√	×	√	√	77.91	70.47
E4 w/o DQ	√	√	×	√	77.67	70.65
E5 w/o L	√	√	√	×	77.97	70.97
F0 RS-CARES	√	√	√	√	77.34	70.43

Note: The structural ablation is conducted under a unified InternImage+CLIP+Mask2Former framework. All ablation variants are trained with the same random seed, optimizer, preprocessing protocol, and training budget. F0 reports the average over three random seeds. This focuses the comparison on CCA, SPLM, dynamic queries, and the language-guided decoding path (LGC+TCA).

**Table 7:** Scale Joint Hit Statistics of Dynamic Query P2/P3 Quotas on the RRSIS-D Validation Set.

P2/P3 Quota	P2 Hit@k2	P3 Hit@k3	Both	P2-only	P3-only	Any-Hit
2/3	58.00%	65.25%	48.16%	9.84%	17.09%	75.09%
3/2	56.56%	60.87%	42.98%	13.58%	17.89%	74.45%
4/1	85.33%	48.22%	46.61%	38.72%	1.61%	86.94%

**Table 8:** Scale Joint Statistics of SPLM for Dynamic Query Candidate Localization on the RRSIS-D Validation Set.

Model	mIoU (%)	P2 Hit@3	P3 Hit@2	P2-only	P3-only	Any-hit
E3 w/o SPLM	58.54	3.34	2.59	2.82	2.07	5.41
F0 (Full)	64.30	52.76	69.62	8.00	24.86	77.62
Delta	+5.76	+49.42	+67.03	+5.18	+22.79	+72.21

## Data Availability

The original RefSegRS data presented in the study are openly available in Hugging Face at https://huggingface.co/datasets/JessicaYuan/RefSegRS or https://doi.org/10.48550/arXiv.2306.08625 (accessed on 14 September 2026). The original RRSIS-D data presented in the study are openly available in reference https://doi.org/10.48550/arXiv.2312.12470 (accessed on 14 September 2026).
